# Preexisting antibodies targeting SARS-CoV-2 S2 cross-react with commensal gut bacteria and impact COVID-19 vaccine induced immunity

**DOI:** 10.1080/19490976.2022.2117503

**Published:** 2022-09-13

**Authors:** Liqiu Jia, Shufeng Weng, Jing Wu, Xiangxiang Tian, Yifan Zhang, Xuyang Wang, Jing Wang, Dongmei Yan, Wanhai Wang, Fang Fang, Zhaoqin Zhu, Chao Qiu, Wenhong Zhang, Ying Xu, Yanmin Wan

**Affiliations:** a Department of Infectious Diseases, Shanghai Key Laboratory of Infectious Diseases and Biosafety Emergency Response, National Medical Center for Infectious Diseases, Huashan Hospital, Fudan University, Shanghai, China; b State Key Laboratory of Genetic Engineering, Institute of Genetics, School of Life Science, Fudan University, Shanghai, China; c Shanghai Public Health Clinical Center, Fudan University, Shanghai, China; d Clinical Laboratory, the First Affiliated Hospital of Zhengzhou University, Key Laboratory of Laboratory Medicine of Henan Province, Zhengzhou, China; e Department of Immunology, School of Basic Medical, Jiamusi University, Jiamusi, China; f Institutes of Biomedical Sciences & Shanghai Key Laboratory of Medical Epigenetics, Fudan University, Shanghai, China; g National Clinical Research Center for Aging and Medicine, Huashan Hospital, Fudan University, Shanghai, China; h Key Laboratory of Medical Molecular Virology (MOE/MOH), Shanghai Medical College, Fudan University, Shanghai, China; i Department of Radiology, Shanghai Public Health Clinical Center, Shanghai, China

**Keywords:** Cross-reactive antibody, SARS-CoV-2, spike protein, commensal gut bacteria, vaccine immunogenicity

## Abstract

The origins of preexisting SARS-CoV-2 cross-reactive antibodies and their potential impacts on vaccine efficacy have not been fully clarified. In this study, we demonstrated that S2 was the prevailing target of the preexisting S protein cross-reactive antibodies in both healthy human and SPF mice. A dominant antibody epitope was identified on the connector domain of S2 (1147-SFKEELDKYFKNHT-1160, P144), which could be recognized by preexisting antibodies in both human and mouse. Through metagenomic sequencing and fecal bacteria transplant, we demonstrated that the generation of S2 cross-reactive antibodies was associated with commensal gut bacteria. Furthermore, six P144 reactive monoclonal antibodies were isolated from naïve SPF mice and were proven to cross-react with commensal gut bacteria collected from both human and mouse. A variety of cross-reactive microbial proteins were identified using LC-MS, of which *E. coli* derived HSP60 and HSP70 proteins were confirmed to be able to bind to one of the isolated monoclonal antibodies. Mice with high levels of preexisting S2 cross-reactive antibodies mounted higher S protein specific binding antibodies, especially against S2, after being immunized with a SARS-CoV-2 S DNA vaccine. Similarly, we found that levels of preexisting S2 and P144-specific antibodies correlated positively with RBD binding antibody titers after two doses of inactivated SARS-CoV-2 vaccination in human. Collectively, our study revealed an alternative origin of preexisting S2-targeted antibodies and disclosed a previously neglected aspect of the impact of gut microbiota on host anti-SARS-CoV-2 immunity.

## Introduction

Antibodies are vital components of the immune system that mediate protection against infections.^1^ When confronting infections, the actual role of preexisting antibody depends on the following features:^2^ high titers of broadly neutralizing antibodies can protect the host against infection. While, when the preexisting antibodies are non-neutralizing or with only a narrow neutralizing spectrum, hosts may not be sterilely protected or only be protected against specific serotypes of viruses. In addition to defending hosts against infections, preexisting antibodies can also impact host immune responses upon infection or vaccination,^3–5^ which is best exemplified by the observations showing that preexisting antibodies shaped the recall immune responses against influenza.^6,7^

For most occasions, preexisting antibodies in adults derive from previous infection or vaccination except some “naturally” produced, poly-reactive antibodies.^2,8^ When encountering a newly emerged or mutated virus, cross-reactive antibodies induced by previously occurred, phylogenetically closely related viruses constitute the main body of the preexisting cross-reactive antibodies. The effect of this kind of preexisting antibodies has been extensively investigated especially for infections of influenza^3,7,9^ and flaviviruses.^10–12^ Of note, previous infection by phylogenetically similar viruses is not the sole source of preexisting cross-reactive antibodies, as it has been clearly clarified that preexisting antibodies against HIV-1 gp41 may stem from exposures to certain commensal gut bacteria.^13–15^ Besides, autoimmune diseases caused by cross-reactivities between microbial and self-antigens also implied that commensal gut bacteria represent important sources of cross-reactive antibodies.^16–19^

Preexisting antibodies against SARS-CoV-2 have also been observed in uninfected healthy individuals, which are speculated to be engendered by previous exposures to human common cold coronaviruses^20–26^ or SARS-CoV.^27–29^ Meanwhile, sequence analyses^30^ and a clinical observation^31^ suggest that preexisting SARS-CoV-2 antibodies might be engendered by common human pathogens and childhood vaccination. Although these two explanations are not mutually exclusive, they both need more experimental evidence to support.

In this study, we found that higher levels of SARS-CoV-2 S2 protein-specific antibodies existed in both healthy human and naïve SPF mice. To track the potential origins of these preexisting cross-reactive antibodies, we mapped and identified a dominant linear antibody epitope on S2, which could be recognized by preexisting antibodies from both healthy human and naïve SPF mice. Monoclonal antibodies (mAbs) against this linear epitope were isolated from naïve SPF mice and proved to cross-react with commensal gut bacteria collected from both healthy human and naïve SPF mouse. Moreover, despite having been discussed iteratively,^32,33^ the influences of preexisting cross-reactive immunities on COVID-19 responses have not been clarified. Here, we showed that high levels of preexisting antibodies did not impair the immunogenicity of a candidate DNA vaccine encoding SARS-CoV-2 spike protein. On the contrary, mice with high levels of preexisting antibodies mounted stronger S2 specific binding antibody responses compared with mice with low levels of preexisting antibodies after immunization with a candidate DNA vaccine.

## Results

### Preexisting antibodies recognizing a dominant linear epitope on SARS-CoV-2 S2 protein were detected in both human and mice

Preexisting antibodies cross-reacted with SARS-CoV-2 S protein have been found in uninfected individuals by multiple previous studies.^22,25,26,34^ It was postulated that the preexisting immunities against SARS-CoV-2 might be induced by previous exposure to seasonal human coronaviruses.^22,32,33,35,36^ However, contradictive evidence suggested that human common cold coronavirus infection did not necessarily induce antibodies cross-reactive with SARS-CoV-2 spike protein.^28,37,38^ In addition to this hypothesis, an alternative explanation suggested that the cross-reactive immunities to SARS-CoV-2 might derive from other common human pathogens and vaccines.^30^

**Figure 1.**
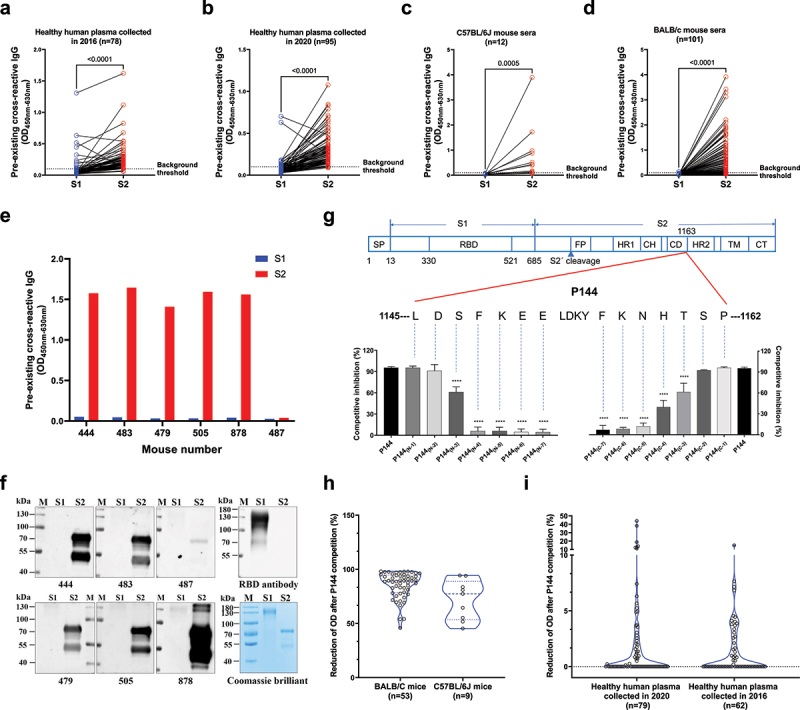
**Preexisting cross-reactive antibodies against a dominant linear epitope on SARS-CoV-2 S2 protein were observed in both healthy human and naïve SPF mice**. (a-d) The preexisting cross-reactive antibodies against S1 and S2 were measured using an in-house ELISA method (Sample dilution factor: 100). (a) Plasma samples of healthy individuals collected in 2016 (n = 78). (b) Plasma samples of healthy individuals collected in 2020 (n = 95). (c) Sera of naïve C57BL/6J mice (n = 12). (d) Sera of naïve BALB/c mice (n = 101). The dotted lines show the threshold of background (3 folds of the average background OD). Statistical analyses were performed using the method of paired t-test. (e and f) The preexisting S1 and S2 reactive antibody levels in the sera of 6 representative mice and WB assays of preexisting cross-reactive antibodies for 6 representative mouse serum samples. Equal amounts of purified S1 and S2 protein (1 μg) were loaded for WB assay. The purities of S1 and S2 proteins were shown by coomassie blue staining. (g) The minimal epitope of P144 was defined using a method of competitive ELISA (Data shown as mean ± SD, n = 5). Purified S2 protein was used as the coating antigen and truncated peptides derived from P144 were used as competitors. The decreases of competitive inhibition reflected the necessity of each amino acid for the epitope recognition. Statistical differences among groups were analyzed using One-way ANOVA. ****, P < .0001. M: molecular weight marker; SP, signal peptide; RBD, receptor-binding domain; FP, fusion peptide; HR, heptad repeat; CH, central helix; CD, connector domain; TM, transmembrane domain; CT, cytoplasmic tail. (h and i) P144 specific binding antibodies were detected using a method of competitive ELISA. The reduction of OD value reflected the presence of P144 binding antibodies. (h) For the detections of P144 specific binding antibodies in naïve SPF mice, purified S2 protein was used as the coating antigen and P144 peptide was used as the competitor. (i) For the detections of P144 specific binding antibodies in healthy individuals, purified BSA-P144 conjugate was used as the coating antigen and P144 peptide was used as the competitor.

To track the origins of the preexisting cross-reactive antibodies to SARS-CoV-2 spike protein, in this study, we first measured the levels of preexisting S protein-specific antibodies in healthy human individuals and SPF mice. Our data showed that the cross-reactive antibody responses against S2 were significantly stronger than those against S1 in plasma samples of healthy human collected both pre (2016 cohort) and post (2020 cohort) the outbreak of COVID-19 pandemic (Figure 1a and 1b). More strikingly, our data showed that binding antibodies targeting S2 could also be detected in two strains of naïve SPF mice (Figure 1c and 1d). And this finding was further confirmed by Western-blotting (WB) assays, which showed that mouse sera with high OD values (Detected by ELISA) (Figure 1e) bound specifically with purified S2 while not S1 (Figure 1f). Quite interestingly, the WB results indicated that cross-reactive antibodies against S2 also existed in the serum of a mouse (#487) with no detectable ELISA binding signal (Figure 1e and 1f). We next performed linear antibody epitope mapping using an in-house developed method of peptide competition ELISA. Our data showed that a single peptide (P144, aa1145-aa1162, 18-mer) accounted for most of the observed preexisting antibody responses toward S2 in mice (Fig. S1). Via employing a series of truncated peptides based on P144, we determined the minimal range of this epitope (1147-SFKEELDKYFKNHT-1160), which locates on the connector domain (adjacent to the N-terminal of HR2 domain) (Figure 1g). We also found that antibodies recognizing this epitope widely existed in both naïve SPF mice and healthy human through competitive ELISA assays (Figure 1h and 1i).

### The P144 specific antibody responses could be engendered by exposures to certain commensal gut bacteria

To explore the potential origins of the preexisted P144-specific antibodies, we first performed phylogenetic analyses among SARS-CoV-2 and other human coronaviruses. The results showed that the aa sequence of P144 was highly conserved among SARS-CoV-2 variants and SARS-CoV, while the similarities between P144 and MERS-CoV or seasonal human coronaviruses were relatively low, especially within the range of predicted antibody binding epitope (boxed fragment) (Fig. S2). The possibility of MHV infection in our SPF-mouse colonies was excluded by the serum screening of MHV specific antibodies (Fig. S3).

**Figure 2.**
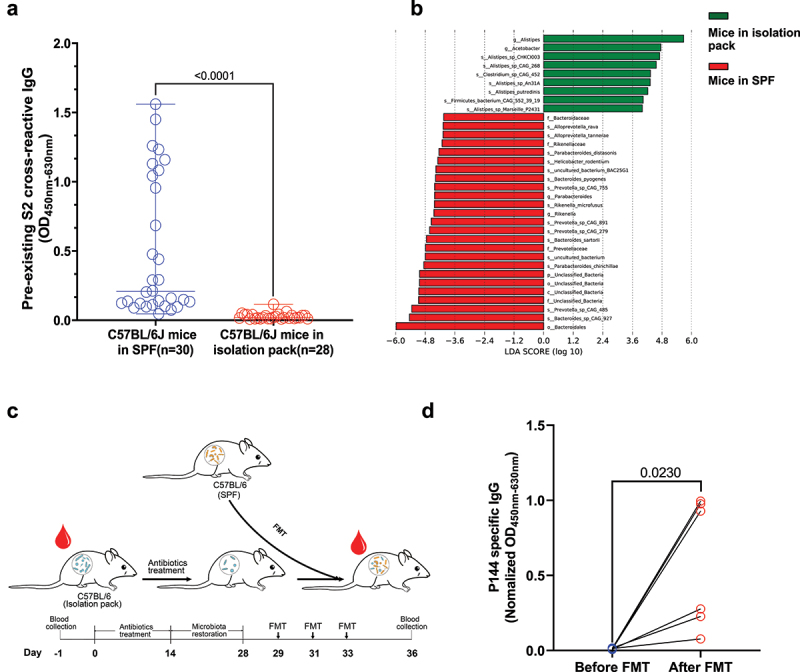
**The presence of the preexisting S2 cross-reactive antibodies was associated with commensal gut bacteria**. (a) Comparison of the levels of S2 reactive antibodies between naïve mice maintained under SPF condition and housed in a sterile isolation pack. (b) Metagenomic analyses of gut microbiota compositions between mice from different housing environments. Differences of bacterial abundance were manifested by linear discriminant analysis (LDA). Only the taxa with an LDA significance threshold > 4 were shown. (c) Schematic overview of the fecal bacteria transplantation (n = 6). The mice bred in a sterile isolation pack were treated with a mixture of ampicillin (1 g/L), metronidazole (0.5 g/L), vancomycin (0.5 g/L) and gentamycin (0.5 g/L) dissolved in drinking water supplemented with D-glucose (36.8 g/L) for 14 d. Two weeks later, fecal bacteria were freshly prepared from SPF mice and delivered to antibiotic treated mice via oral gavage. (d) Comparison of P144 reactive antibodies in mouse sera collected before and after FMT. The OD value of P144 binding antibody was normalized to the concentration of total IgG for each mouse. Data are shown as mean ± SD (n = 6). Statistical analyses were performed by the method of paired t-test. (p), Phylum. (c), Class. (o), Order. (f), Family. (g), Genus. (s), Species.

Subsequently, to investigate whether environmental factors contribute to the induction of these S2 cross-reactive antibodies, we compared the levels of preexisting S2 binding antibodies between C57BL/6J mice housed in SPF condition and C57BL/6J mice maintained in a sterile isolation pack. Our data showed that the levels of preexisting S2 binding antibodies were significantly higher in SPF mice (Figure 2a). Through metagenomic sequencing, we further demonstrated that the compositions of commensal gut bacteria were significantly different between mice housed in different environments (Fig. S4A). The abundance of family *Bacteroidaceae*, family *Prevotellaceace* and genus *Parabacteroides* increased significantly in the commensal gut bacteria of SPF mice (Figure 2b). Moreover, frequencies of memory B cells measured by B cell ELISPOT (Fig. S4B) and frequencies of S2 specific B cells (CD45^+^CD19^+^S2^+^) measured by flowcytometry (Fig. S4C and S4D) were significantly higher in mesenteric lymph nodes (MLNs) than those in spleens of mice with preexisting S2 binding antibodies. Consistently, via 16s rDNA sequencing, we found that the gut microbiota compositions of SPF mice with different levels of preexisting S2 binding antibodies might be different (Fig. S5). To further clarify the role of gut microbiota in the induction of S2 cross-reactive antibodies, mice fed in a sterile isolation pack were transplanted with fecal bacteria prepared from SPF mice (Figure 2c). We found that the abundances of P144 reactive antibodies in mouse sera significantly increased after fecal microbiota transplantation (FMT) (Figure 2d). These results collectively suggested that the S2 cross-reactive antibodies could be induced by exposures to certain microbial antigens.

### P144 specific monoclonal antibodies reacted with commensal gut bacteria of both human and mouse and showed limited neutralizing activities

To probe the potential antigens that might induce the P144 binding antibodies, we isolated 6 mAbs from two naïve SPF mice (one C57BL/6J mouse and one BALB/c mouse) with high levels of preexisting S2-specific antibody responses. The results of microscale thermophoresis (MST) assays showed that the binding affinities with S2 protein were similar among the 6 mAbs (F5, 2.07 μM; H9, 0.98 μM; E10, 3.53 μM; G13, 2.10 μM; M3, 1.46 μM; G18, 2.25 μM) (Fig. S6). Five of these mAbs recognized P144 solely (Fig. S7A), while one mAb (clone M3) bound with P144 and P103 simultaneously (Fig. S7B). Results of competitive ELISA showed that the minimal epitopes varied slightly among the five P144-specific mAbs, especially at the C-terminal of P144 (Fig. S7A). The neutralizing potentials of these isolated monoclonal antibodies were evaluated using a pseudo-virus-based neutralization assay. Our results showed that these monoclonal antibodies exhibited limited neutralizing activity against 5 SARS-CoV-2 variants (Fig. S8), which might be partially explained by their relatively low affinities to S2 protein (Fig. S6). We also evaluated the activities of the mAbs in inhibiting spike protein mediated cell fusion according to a previously described method.^39^ The data showed that 6 mAbs significantly reduced syncytium formation at concentration of 10 μg/ml as compared to the IgG (Fig. S9).

**Figure 3.**
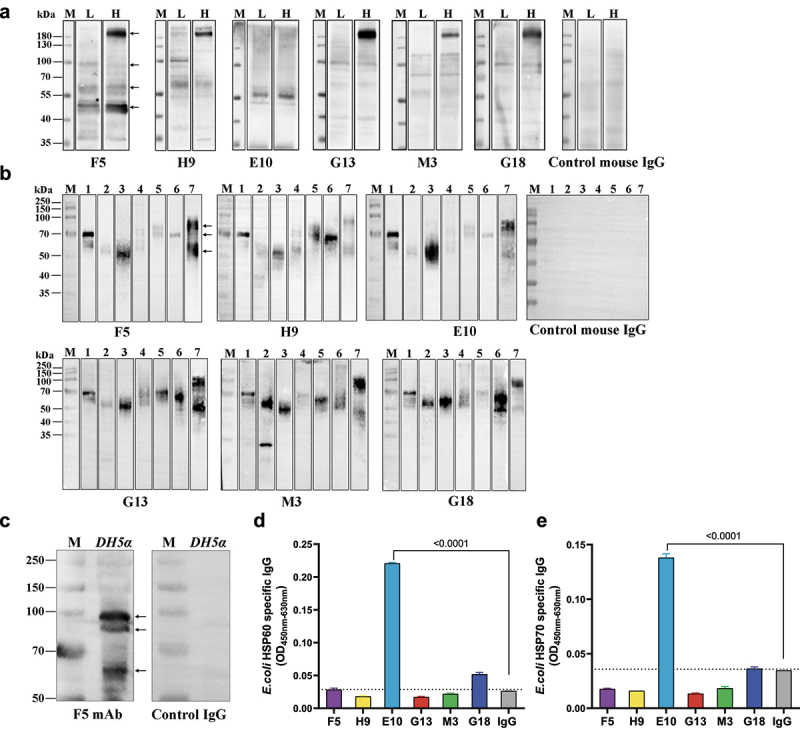
**P144 binding mAbs isolated from naïve SPF mice reacted with commensal gut bacteria from both human and mouse**. Reactivities between P144 binding mAbs and gut microbial antigens were detected using WB assays. A purified mouse IgG was used as the control. (a) WB assays of mouse fecal bacteria samples. L: mixed fecal bacteria samples collected from 3 mice with low levels of preexisting S2 binding antibodies (OD_450nm-630nm_≤0.140, 1:100 diluted); H: mixed fecal bacteria samples collected from 3 mice with high levels of preexisting S2 binding antibodies (OD_450nm-630nm_≥0.615, 1:100 diluted). (b) WB assays of fecal bacteria samples collected from 7 healthy individuals (Lanes 1–7). All the mAbs and the control mouse IgG were tested at the final concentration of 1 μg/ml. Black arrows indicate the locations of protein bands selected for the mass spectrometry analysis. (c) Reactivity between a P144 specific mAb (clone F5) and *E. coli* was validated by WB. F5 or a purified mouse IgG was used as the first antibody at the concentration of 1 μg/ml. Black arrows point out the bands with MWs equal to *E. coli* proteins identified by LC-MS analysis. (d) Recognition of purified *E. coli* HSP60 protein by P144 reactive antibodies. (e) Recognition of purified *E. coli* HSP70 protein by P144 reactive antibodies. All the mAbs and control IgG were measured at a final concentration of 10 μg/ml. Dotted line represents the mean OD value of control IgG plus 2-fold SD (mean+2SD).

To prove the cross-reactivities between S2 and commensal gut microbial antigens, whole cell lysates (WCL) of mouse and human commensal gut bacteria were prepared and used as antigens for WB assays, respectively. As shown in Figure 3a, specific bindings with the WCL of mixed fecal bacteria prepared from mice either with low levels of preexisting antibodies (L) or with high levels of preexisting antibodies (H) could be clearly visualized for each isolated mAbs. It was noteworthy that all mAbs except E10 strongly recognized a band around 180KD in the sample from mice with high preexisting antibody responses. E10 predominantly recognized a band around 55KD in both samples, while stronger binding with the sample from mice with high preexisting antibody responses could be visually observed (Figure 3a). Among the six mAbs, F5 showed the most diverse binding capacity. In addition to the band around 180KD, F5 bound with a band around 55KD (similar with E10) and a band between 40KD-55KD (Figure 3a). In comparison with the WB results of mouse samples, the recognized bands were less consistent across different human fecal bacteria samples (Figure 3b), presumably due to the individual to individual variation of gut microbiota composition. We found that a band around 70KD was recognized by most mAbs in 4 (lanes 1, 5, 6, 7) out of 7 samples and a band between 50KD-70KD was recognized by all mAbs in 3 (Lanes 2, 3, 7) out 7 samples. Our data showed that the recognition patterns toward each fecal bacteria sample were generally stable across different mAbs except E10 (Figure 3a and 3b). Then, we performed V(D)J gene sequencing for the 6 antibody clones. Our data showed that the same VH/VL gene combination (IGHV2-5*01, IGKV6-23*01) is used by 4 clones (F5, G18, H9 and M3) (Table S1). Interestingly, among these 4 clones, F5 and M3 were isolated from a C57BL/6J mouse, while G18 and H9 were isolated from a BALB/c mouse, suggesting it might be a public antibody shared by different mice. G13 uses the same VH gene as the above 4 mAbs in combination with a different VL gene (IGLV2*02). The VH/VL gene usage (IGHV5-9*02, IGKV8-30*01) of E10 is completely different from other clones, which might explain its unique WB patterns shown in Figure 3a and 3b.

Proteins corresponding to specifically recognized bands were excised from Coomassie blue-stained gels and analyzed by the mass spectrometry. For the mouse fecal bacteria samples, protein bands with molecular weights around 180KD, 100KD, 55KD-70KD and 40KD-55KD (indicated by arrows in Figure 3a, panel F5) were selected. For human fecal bacteria samples, protein bands with molecular weights around 50KD-70KD, 70KD and 70KD-100KD (indicated by arrows in Figure 3b, panel F5) were selected. The lists of proteins identified in mouse and human samples were shown in Table S2 and Table S3, respectively. Proteins with molecular weights corresponding approximately to the excised protein bands were identified for both human and mouse fecal bacteria samples. Of note, multiple proteins within the theoretical MW range of 58KD to 60KD were found to be identical between mouse and human samples, which included Fumarate hydratase class I (Accession# P14407), Formate-tetrahydrofolate ligase OS (Accession# Q189R2), Phosphoenolpyruvate carboxykinase (ATP) OS (Accession# C4ZBL1 and A6LFQ4) and 60 kDa chaperonin OS (Accession# A0Q2T1). To verify the cross-reactivity of the proteins detected by LC-MS, we selected *E. coli* (DH5α strain) as a representative target because *E. coli* derived Fumarate hydratase class I (Accession# P14407) were found in both human and mouse fecal bacteria samples. The result of WB assay showed that the P144-specific mAb (Clone F5) recognized multiple bands with MWs consistent with *E. coli* proteins identified by LC-MS (Figure 3c). Employing an in-house ELISA method, we further showed that one of the isolated mAbs (E10) bound specifically with purified microbial HSP60 and HSP70 proteins (Figure 3d and 3e), and antibodies in sera of vaccinated mice and humans also bound these proteins (Fig. S10). However, no obvious binding was observed between E10 and human HSP60 or HSP70 (Fig. S11).

### Preexisting S2 cross-reactive antibodies impacted specific immunities induced by a candidate COVID-19 DNA vaccine in mice

**Figure 4.**
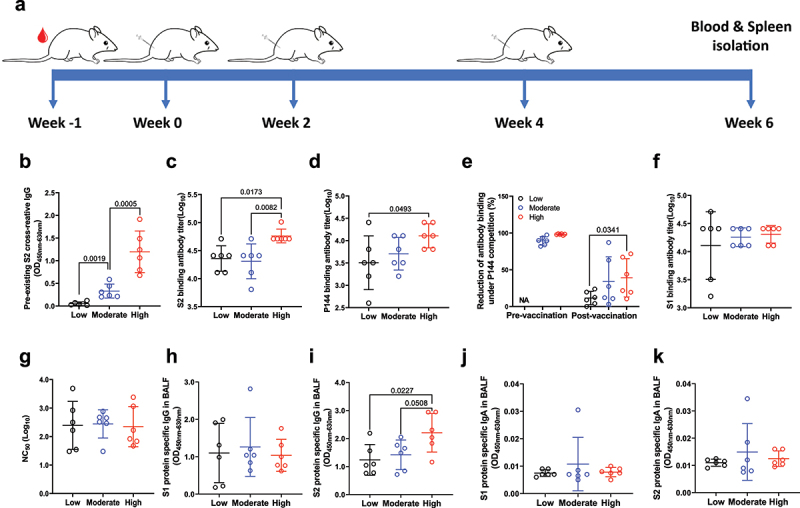
**Impacts of preexisting antibodies on the humoral immune responses elicited by a DNA vaccine encoding SARS-CoV-2 S protein**. (a) Schematic illustration of the experiment schedule. 50 μg of the DNA vaccine was injected intra muscularly into each mouse at week 0, week 2 and week 4, respectively. Two weeks after the final vaccination, the mice was euthanized for the measurements of specific immune responses. (b) Peripheral blood of mice housed in SPF was collected before immunization and levels of preexisting S2 specific antibodies were measured by ELISA. Based on the levels of preexisting S2 binding antibodies, the mice were divided into three groups: low (0.015< OD_450nm-630nm_≤0.130, n = 6), moderate (0.130< OD_450nm-630nm_≤0.750, n = 6) and high (OD_450nm-630nm_>0.750, n = 6). (c) Endpoint IgG titers against S2 were compared at 2 weeks post the last immunization. (d) Comparisons of P144 specific IgG titers as measured using BSA-P144 conjugate as the coating antigen. (e) Comparisons of P144 specific binding antibody levels as determined using a method of competitive ELISA. Purified S2 protein was used as the coating antigen and P144 peptide was used as the competitor. The reduction of OD value reflected the presence of P144 binding antibodies. (f) Endpoint IgG titers against S1 measured at 2 weeks post the final vaccination. (g) Neutralizing antibody titers against SARS-CoV-2 D614 G pseudo-virus in serum of mice at 2 weeks post the final immunization. BALF was collected from each mouse after euthanasia. Specific IgG (h and i) and IgA (j and k) against S1 or S2 were detected using in-house ELISA methods. All the BALF samples were adjusted to the initial total protein concentration of 51.9 μg/ml. Data are shown as mean ± SD, n = 6. Statistical analyses were performed by the method of one-way ANOVA.

Preexisting antibodies has been shown to be able to shape the recall immune responses upon influenza infection and vaccination.^6^ And the concern about how the preexisting immunities may influence the effect of a SARS-CoV-2 vaccine has also attracted lots of attention.^33^ To investigate the impact of the preexisting P144 antibodies on the immunogenicity of a candidate DNA vaccine, 18 BALB/c mice were divided into 3 groups according to their levels of preexisting S2 binding antibodies and immunized with a DNA vaccine encoding the full length of SARS-CoV-2 S protein (Figure 4a and 4b). Our data showed that mice with high levels of preexisting S2 binding antibodies mounted significantly higher S2 binding antibody responses after vaccination compared to mice with low or moderate levels of preexisting S2 binding antibodies (Figure 4c). The average level of P144 specific antibody responses was also stronger in mice with high levels of preexisting S2 binding antibodies than that of mice with low preexisting S2 binding antibody responses (Figure 4d and 4e). By comparison, both the S1 binding antibody (Figure 4f) and the neutralizing antibody titers (Figure 4g) did not significantly differ among all groups, despite that mice with moderate or high levels of preexisting antibodies tended to mount higher average titer of S1 binding antibodies. We further investigated the influence of preexisting antibodies on humoral immune responses in mouse respiratory tract after vaccination. And our data showed that the levels of S1-specific IgG in BALF were similar among the three groups after DNA vaccination (Figure 4h), while the average level of S2-specific IgG in BALF from mice with high preexisting S2 binding antibodies was significantly higher than those from mice with low preexisting antibodies (Figure 4i). S protein specific IgA responses were comparable among the three groups (Figure 4j and 4k).

In addition to antibody measurement, we compared S protein-specific T cell responses among the three groups as well (Fig. S12A). The results showed that the candidate DNA vaccine elicited robust S protein-specific T cell responses in all groups. Although no statistical significance was reached, interesting trends were observed: first, mice with high levels of preexisting S2 binding antibodies tended to mount relatively stronger S1 and S2-specific IFN-γ^+^ T cell responses (Fig. S12B); second, as measured by the releases of IL-6, IL-2 and TNF-α, mice with high levels of preexisting antibodies tended to mount stronger T cell responses against both S1 and S2 (Fig. S12C and S12D). The major findings of this part of the study were validated by a repeated experiment (Fig. S13).

### The impact of preexisting antibodies on the recognition of P144 epitope after vaccination

**Figure 5.**
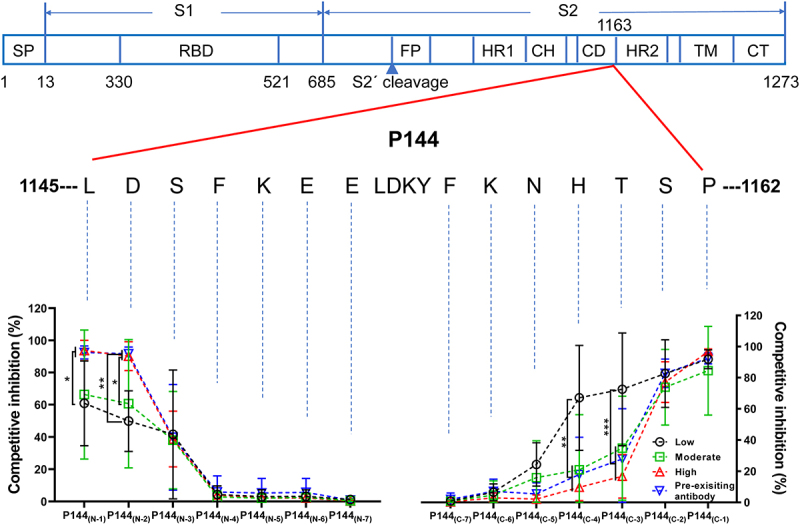
**The impact of preexisting antibodies on the recognition of P144 after vaccination**. The minimal epitope recognized by mouse sera after vaccination was analyzed using a method of competitive ELISA. Purified BSA-P144 conjugate was used as the coating antigen and truncated peptides derived from P144 were used as the competitors. The decreases of competitive inhibition reflected the necessity of each amino acid for the epitope recognition. Statistical analyses were performed by the method of two-tailed t-test (*, P < .05, **, P < .01, ***, P < .001).

As the preexisting antibodies in naïve SPF mice predominantly recognized P144 (Figure 1g and Fig. S1), we delineated the impact of preexisting antibodies on the recognition of this epitope after vaccination. Our results showed that the minimum epitope recognition pattern by the sera of mice with high levels of preexisting antibodies remained unchanged after vaccination (Figure 5). Whereas the minimum epitope recognized by the sera of mice with moderate and low levels of preexisting antibodies altered at either the N-terminal or both terminals of P144 (Figure 5).

### Preexisting S2 cross-reactive antibodies correlated with RBD binding antibody responses after two-dose inactivated SARS-CoV-2 vaccination

**Figure 6.**
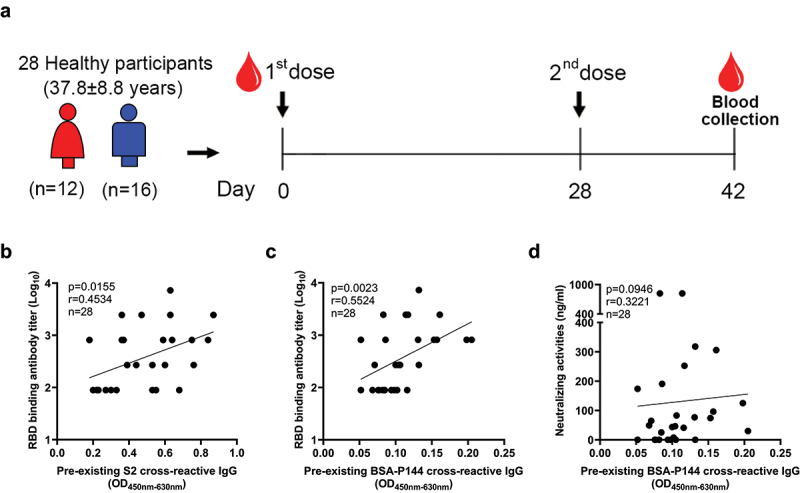
**Levels of preexisting S2 cross-reactive antibodies correlated with RBD-binding antibody responses elicited by an inactivated SARS-CoV-2 vaccine in human**. (a) Peripheral blood samples were collected from 28 healthy individuals who were vaccinated with two doses of an inactivated SARS-CoV-2 vaccine (BIBP-CorV) at baseline and 14 d post the 2^nd^ dose, respectively. The RBD binding antibody titers were measured by ELISA. The neutralizing antibody responses were quantified by a commercialized surrogate virus neutralization test (sVNT) (Suzhou Sym-Bio Life Science Co., Ltd). (b and c) Correlations between RBD binding antibody titers and levels of preexisting S2 or P144 specific IgG. (d) Correlation between neutralizing antibody concentrations and preexisting P144 binding antibody levels. Statistical analyses were performed using the method of Spearman’s correlation. The experiment was repeated twice.

To investigate how the preexisting cross-reactive antibodies may influence the COVID-19 vaccine induced immunity, peripheral blood samples were collected from 28 healthy individuals who received two doses of an inactivated SARS-CoV-2 vaccine (Figure 6a). Correlation analyses showed that both the OD values (Figure 6b and 6c) and the titers (Table S4) of preexisting S2 and P144 specific antibodies were significantly associated with RBD binding antibody titers at 14 d after immunization. Additionally, although not statistically significant, the preexisting P144 binding antibody levels tended to correlate positively with neutralizing antibody responses after vaccination (P = .0946) (Figure 6d).

## Discussion

The origins of preexisting cross-reactive immunities against SARS-CoV-2 have been investigated vigorously since the outbreak of the pandemic.^40^ Accumulating data suggest that cross-reactive T cells^33,41–44^ in SARS-CoV-2 unexposed human might be induced by previous infections of other hCoVs. While the origins of preexisting cross-reactive antibodies could not be completely explained by previous infections of other coronaviruses, as recent studies revealed that the magnitude of antibody responses to SARS-CoV-2 S protein in the sera of patients with COVID-19 was not related to HCoVs’ S titers^45^ and immunization with coronaviruses OC43 did not induce significant SARS-CoV-2 S protein cross-reactive antibodies in mice. Moreover, it has also been observed that SARS-CoV-2 S protein specific binding antibody responses were weak in SARS-CoV-2 unexposed individuals with obvious binding antibody responses against S proteins of common cold hCoVs.^20,46^

To track the potential origins of the preexisted cross-reactive antibodies targeting SARS-CoV-2 spike protein, in this study, we first screened the cross-reactive antibody responses in SARS-CoV-2 unexposed human plasma collected in 2020 and 2016, respectively. In both cohorts, we found that the magnitudes of S2 binding antibodies were significantly higher than those of S1 binding antibodies. This finding is consistent with previous studies showing that preexisting S2 cross-reactive antibody responses are stronger than S1 cross-reactive antibody responses in SARS-CoV-2 unexposed individuals.^26,45,47,48^ Since S2 cross-reactive antibody responses have also been observed in unexposed animals,^45^ we continued to screen the cross-reactive antibody responses in two strains of naïve SPF mice. Our data showed that the OD values of S2 cross-reactive antibodies were significantly higher than those of S1 cross-reactive antibodies in naïve BALB/c and C57BL/6J mice.

To facilitate the search of potential antigens that induced the cross-reactive antibodies, we identified a dominant antibody epitope (P144) through a method of competitive ELISA-based linear antibody epitope mapping. P144 is located within the connector domain of S2 (aa1147-aa1160, directly N-terminal of the HR2 region). The same epitope has been predicted^30^ and detected in both SARS-CoV-2 unexposed and infected individuals by multiple previous studies.^22,23,25,26,32^ In this study, P144-specific antibody responses were detected in plasma samples of healthy individuals collected in both 2020 and 2016. More interestingly, we found that the preexisting S2 cross-reactive antibodies in naïve SPF mice were predominantly against this epitope. Of note, although we cannot rule out the possibility that the P144-targeted antibodies might be induce by prior exposures to common cold coronaviruses in human, the sequence similarities between P144 and four seasonal hCoVs are relatively low. And it has also been shown that this epitope was more frequently recognized than its homologous peptides from common cold hCoVs by antibodies of COVID-19 negative individuals.^23^ These evidences collectively implied that the preexisting S2-specific antibodies might not be necessarily elicited by previous common cold coronavirus infections in human.

To unveil the origin of the preexisting S2 binding antibodies in mice, we first measured S2-specific B cells by B cell ELISPOT and flowcytometry and found that the frequency of S2-specific B cells was significantly higher in mesenteric LN than in spleen, suggesting that the gastrointestinal tract might be the primary site where the cross-reactive B cells were activated. As gut bacteria can promote B cell diversification and stimulate antibody production in both T-dependent and -independent ways,^49^ we speculated that exposure to certain gut microbial antigens might account for the presence of the cross-reactive antibodies. To prove this hypothesis, we interrogated the relationship between the levels of preexisting S2 cross-reactive antibodies and the compositions of mouse gut microbiota. Our results showed that mice with different gut microbiota composition had different levels of preexisting S2 cross-reactive antibodies (Fig. S5). Moreover, we found that transplantation of fecal bacteria isolated from SPF mice could induce S2 reactive antibodies in mice bred in a sterile isolation pack. The above evidence suggested that the S2 reactive antibodies could be induced by exposure to certain commensal gut bacteria.

We are not able to define the bacterial strains contributing to the induction of P144 reactive antibodies in the current study, because it is technically hard to get pure cultures for each potential strain. Instead, we tried to identify potential microbial antigens that may induce the cross-reactive antibodies. To do so, we isolated six P144-specific monoclonal antibodies from a naïve BALB/c mouse and a naïve C57BL/6J mouse, respectively. All the six mAbs were confirmed to be able to bind with P144 and showed weak neutralizing capacities against five SARS-CoV-2 variants. This observation is slightly different from few previous studies, which find antibodies targeting this epitope display broadly neutralizing activities.^50–52^ A possible explanation might be that somatic hypermutation levels of our antibodies are low, which may limit their binding affinities. As a previous study suggests that an mAb targeting this epitope inhibits SARS-CoV-2 mediated cell fusion,^50^ we measured the activities of P144 reactive mAbs in blocking spike protein mediated cell fusion, and confirmed that these mAbs significantly reduced cell fusion compared with a control IgG. Leveraging these mAbs, we detected the cross-reactive antigens in mouse and human fecal microbiota through WB assays. Compared with a control mouse IgG, specific bands were observed for each mAb, which manifested the antibody cross-reactivities between SARS-CoV-2 and commensal gut bacteria. This observation is corroborated by a previous study showing that immunization with this peptide-induced antibodies cross-reacted with human gut bacteria.^53^ The strongly recognized protein bands were further analyzed by LC-MS. Our data showed that cross-reactive antigens derived from *Bacteroides* and *Parabacteroides* were frequently identified in fecal bacteria samples of both human and mouse, which was consistent with our metagenomic sequencing data showing that the abundance of *Bacteroides* and *Parabacteroides* was significantly higher in the commensal gut bacteria of mice with high preexisting S2 binding antibody levels. More intriguingly, five cross-reactive microbial antigens were identified in mouse and human fecal samples simultaneously, implying that the S2 cross-reactive antibodies might naturally occur in different species of mammals.

According to our LC-MS results, few proteins of *E. coli* and HSP60/HSP70 proteins of multiple bacterial strains can be recognized by P144 reactive antibodies. These results were verified by experiments showing that P144 reactive mAbs could bind with the lysate of an *E. coli* strain (DH5α) and purified HSP60/HSP70 proteins. However, due to the limited availabilities of pure cultures of commensal gut bacteria and their protein derivatives, we were not able to verify all the potential reactive strains in this study. Alternatively, we tried to refine our LC-MS results through sequence similarity analysis. We found that P144 shared varied identities with LC-MS captured proteins (40%–70%, length ≥8 aa residues). The similarities are not high enough to support confident identifications of potential cross-reactive epitopes based on our current data. To clarify this issue, we plan to expand experimental screening through collaboration in future.

In parallel with tracking the initial antigens that induced the S2 cross-reactive antibodies, we investigated the impact of preexisting antibodies on the immunogenicity of a candidate DNA vaccine as well. According to previous reports, preexisting cross-reactive antibodies may influence the effects of different vaccines differentially.^6,54^ In this study, we found that the preexisting cross-reactive antibodies shaped the vaccine-induced immune responses in both mouse and human. Mice with high levels of preexisting antibodies mounted stronger S2 binding antibodies in both peripheral blood and bronchial lavage after vaccination. More interestingly, we found that the preexisting antibody levels correlated positively with post-vaccination RBD binding antibody titers in human. These findings demonstrated that the preexisting S2 binding antibodies could facilitate the generation of vaccine induced antibody responses. Through epitope mapping, we observed that the preexisting antibodies strongly restricted the minimal epitope recognition in mice with high levels of preexisting antibodies, which suggested that the imprint effect of preexisting cross-reactive antibodies on vaccine induced antibody responses was primarily epitope-specific. In addition to antibody response, we also found that the high levels of preexisted S2 binding antibodies tended to improve specific T cell responses induced by SARS-CoV-2 S DNA vaccine. Since we did not perform the live virus challenge, it is still not clear how the preexisting S2 cross-reactive antibodies will impact vaccine efficacy *in vivo*. Nonetheless, as both our results and a recently published study suggested that antibodies targeting P144 epitope could neutralize SARS-CoV-2,^50–52^ we speculate that the preexisting P144 cross-reactive antibodies may have protective effect. In this part of the study, we did not test the influences of preexisting antibodies on vaccine elicited immunities through passive antibody transfer, because: First, passive antibody transfer cannot generate S2 reactive memory B cells in recipient mice, which was observed in the mesenteric lymph nodes and spleens of mice with preexisting S2 cross-reactive antibodies (Fig. S4). Second, it was technically difficult to maintain relatively stable *in vivo* antibody level for a long term via passive transfer.

Two major limitations should be noted for this study. First, we are unable to conduct a live-virus challenge experiment because of the difficulties to acquire mouse adapted SARS-CoV-2 virus and to get access to an ABSL-3 lab. Hence, it is not clear how the preexisting S2 cross-reactive antibodies will impact vaccine efficacy *in vivo*. Second, we are not able to define the bacteria that contribute to the induction of P144 reactive antibodies in the current study, we plan to tackle this issue in near future. Despite of these limitations, we provided evidence showing that antibodies targeting a conserved linear epitope on S2 cross-reacted with gut microbial antigens from both human and mouse, manifesting that some of the preexisting cross-reactive antibodies might be induced by exposure to certain commensal gut bacteria. These preexisting antibodies hold the potential to block SARS-CoV-2 infection and can enhance the S2-specific antibody responses elicited by a DNA vaccine in a mouse model. A deep understanding of preexisting cross-reactive antibodies against SARS-CoV-2 will enable better therapeutic, diagnostic and vaccine strategies. Further investigations into the functions of P144 cross-reactive antibodies may assist in delineating the role S2-specific antibody upon SARS-CoV-2 infection and elucidating the mechanisms underlying the gastrointestinal symptom caused by COVID-19.^55–57^

## Materials and methods

### Ethics statement

All experiments and methods were performed in accordance with relevant guidelines and regulations. Experiments using mice and samples of healthy human were approved by the Research Ethics Review Committee of the Shanghai Public Health Clinical Center Affiliated to Fudan University.

### Plasma samples of healthy human

Two batches of plasma samples were collected from healthy individuals at the health screening clinic of Shanghai Public Health Clinical Center. A concurrent batch was collected in December 2020. All the 95 individuals enrolled in this batch reported no epidemiological link with confirmed COVID-19 patients and were confirmed to be free from any chronic or acute disease. Viral RNA tests confirmed that all individuals in this batch were free from SARS-CoV-2 infection. In addition, a historical batch of 78 plasma samples from healthy individual cohort (collected in 2016) were also measured for their cross reactivities with SARS-CoV-2 S protein. As the local prevalence in Shanghai was extremely low during previous SARS-CoV-1 epidemic, we did not do the serum screening for previous SARS-CoV-1 infection. Instead, we collected the information regarding previous SARS-CoV-1 infection status through either questionnaire survey (for the 2020 cohort) or telephone follow-up (for the 2016 cohort). There is no self-reported previous SARS-CoV-1 infection among the two cohorts. Demographical information about these two cohorts was described in Table S5.

### Screening of mouse hepatitis virus (MHV) infection in mice

A commercialized kit (Cat#SY-M02196, Shanghai Shuangying Biotechnology Co., Ltd, China) for detecting MHV specific antibodies was used to monitor MHV infection following the manufacturer’s instruction. Briefly, the serum of mice was diluted at 1:5 and added into plate. Meanwhile, the positive and negative control reagent were also added into plate. Subsequently, HRP-conjugate reagent was added into each well and incubated for 1 h at 37°C. After the last wash, both chromogen solution A and chromogen solution B were added and incubated for 15 min at 37°C. Finally, the stop solution was added and mixed. The values of optical density at OD_450nm_ and OD_630nm_ were measured using 800 TS microplate reader (Cat# 800TS, Biotek, USA) within 15 minutes.

### Detection of SARS-CoV-2 S1 and S2 specific binding antibodies

In-house enzyme-linked immunosorbent assays (ELISA) were developed to measure SARS-CoV-2 S1 and S2 specific binding antibodies. High-binding 96-well EIA plates (Cat# 9018, Corning, USA) were coated with purified SARS-CoV-2 S1 (Cat# 40591-V08H, Sino Biological, China), S2 proteins (Cat# 40590-V08B, Sino Biological, China), recombinant *E. coli* HSP60 (Cat#HSP-004, Prospec, Jsrael), *E. coli* HSP70 (Cat#HSP-006, Prospec, Jsrael), human HSP60 (Cat#HSP-016, Prospec, Jsrael) or human HSP70 (Cat#HSP-170, Prospec, Jsrael) at a final concentration of 1 µg/ml in carbonate/bi-carbonate coating buffer (30 mM NaHCO_3_,10 mM Na_2_CO_3_, pH 9.6). Subsequently, the plates were blocked with 1× PBS containing 5% milk for 1 hour at 37°C. Next, 100 μl of diluted human plasma, mouse serum or mAbs was added to each well. After 1-hour incubation at 37°C, the plates were washed with 1× PBS containing 0.05% Tween 20 for 5 times. Then, 100 μl of a HRP labeled rabbit anti-human IgG antibody (Cat# ab6759, Abcam, UK) or goat anti-mouse IgG antibody (Cat# 115–035-003, Jackson Immuno Research, USA) diluted in 1× PBS containing 5% milk were added to each well and incubated for 1 hour at 37°C. After a second round of wash, 100 μl of TMB substrate reagent (Cat# MG882, MESGEN, China) was added to each well. 15 minutes later, the color development was stopped by adding 100 μl of 1 M H_2_SO_4_ to each well and the values of optical density at OD_450nm_ and OD_630nm_ were measured using 800 TS microplate reader (Cat# 800TS, Biotek, USA). Reproducibility of the ELISA assay was validated by repeated measurement of the same panel of human sera.

### Competitive ELISA

According to the reference sequence of SARS-CoV-2 (Genebank accession number: NC_045512), peptides (18-mer overlapping by 11 residues, purities > 95%) encompass the full length of S protein were synthesized by GL Biochem (Shanghai, China). BSA coupled with P144 (BSA-P144) was also synthesized and purified by GL Biochem (Shanghai, China) (Purity > 95%). The experiment procedure was generally similar with the afore mentioned in-house ELISA assays, except that the diluted mouse serum or human plasma were incubated with synthesized peptides (5 μg/ml) or a non-relevant peptide (OVA_323-339_) for 1 hour at room temperature before adding into the coated EIA plates.

### FACS analysis of S2 specific B cells in mice

Spleen and mesenteric lymph nodes were isolated from naïve SPF mice and single-cell suspensions were prepared. After counting, 1 × 10^6^ single cells were resuspended in PBS and stain with Live/dead Zombie Aqua Fixable viability Dye (Cat#423101, Biolegend, USA) for 15 min at room temperature. After incubation, the cells were washed 500 µl R10 (RPMI1640 containing 10% fatal bovine serum) and then incubated with biotinylated S1 protein (Cat# 40591-V08H-B, Sino Biological, China) or biotinylated S2 protein (Cat# 40590-V08B-B, Sino Biological, China) for 30 minutes at 4°C. After incubation, the cells were washed twice with 500 µl R10. Then, the cells were incubated with the mixture of PE-anti-mouse CD19 (Cat# 152408, Biolegend, USA, 1 µl/test), BV785-anti-mouse CD45 (Cat# 103149, Biolegend, USA, 1 µl/test) and Streptavidin-IF647 (Cat# 46006, AAT Bioquest, USA, 0.2 µl/test) at 4°C for 30 minutes. After washing, the stained cells were resuspended in 200 µl 1× PBS and analyzed using a BD LSRFortessa™ Flow Cytometer. The data were analyzed using the FlowJo software (BD Biosciences, USA).

### Preparation of P144 specific monoclonal antibodies

Monoclonal antibodies against P144 were prepared from one naïve BALB/c mouse and one naïve C57BL/6J mouse respectively using the hybridoma technique. Briefly, freshly isolated splenocytes were mixed and fused with SP2/0 cells at a ratio of 1:10. Hybridoma cell clones secreting P144 specific antibodies were screened by ELISA and monoclonal hybridoma cells were selected by multiple rounds of limited dilution. Selected clones of hybridoma cells were injected intraperitoneally into BALB/c× ICR hybrid mice. About 1–2 weeks later, peritoneal fluid was collected, and monoclonal IgG was purified using Protein A resin. The purities of monoclonal antibodies were verified using SDS-PAGE and the antibody concentrations were determined using a BCA kit (Cat# P0012, Beyotime Biotechnology, China).

### V(D)J gene sequencing of P144 reactive monoclonal antibodies

The V(D)J genes of P144 reactive monoclonal antibodies were sequenced by AZENTA life science. Briefly, total RNA was extracted from hybridoma cells using Trizol reagent (Cat# R4801-02, Invitrogen, USA). 5` RACE was performed with SMARTer RACE cDNA Amplification Kit (Cat# 634923, Clontech, USA), total RNA input was 500–2000 ng. V(D)J genes of heavy and light chains were amplified by PCR. The PCR products were purified through gel extraction using a QIAquick Gel Extraction Kit (Cat# 28704, Qiagen, USA). NGS libraries were constructed by using VAHTS Universal DNA Library Prep Kit for Illumina (Cat# ND607, Vazyme, China). The qualified libraries were sequenced on the Illumina Miseq 2 × 300 platform (Illumina, San Diego, CA, USA). Raw fastq files were first subject to quality assessment. Adapters and bases with poor quality scores (Q value lower than 20) were removed using Trimmomatic (v0.36) to generate clean data (trimmed data). Pandaseq (2.10) was used to merge pair-end read. Merged sequences were processed by IgBLAST software to identify the V(D)J sequences. The reference sequences were obtained in IMGT database (IMGT, https://www.imgt.org/).

### Isolation of gut commensal bacteria and preparation of whole cell lysate (WCL)

About 2 g of each fecal sample was suspended with 15 ml sterile 1× PBS and vortexed thoroughly to obtain uniform mixtures. After centrifugation at 200 × g for 5 min, the supernatants were collected, and the sediments were discarded. This process was repeated twice. Next, all the supernatant samples were centrifuged twice at 9000 × g for 5 min and the supernatants were discarded. The precipitated bacteria pellets were resuspended in 500 µl of 1× PBS (containing 1 mM PMSF) and disrupted with an ultrasonic cell crusher (the probe-type sonicator, Model JY92-II; Ningbo Scientz Biotechnology Co., Ltd, China). After sonication, the samples were centrifuged at 10000 rpm for 30 minutes to remove the cellular debris.

### Western blotting

WCL containing 10 μg of total protein was separated by SDS-PAGE (10% acrylamide gels) and then transferred onto a PVDF membrane (Cat# IPVH00010, Millipore, USA) or stained with Coomassie brilliant blue. After blocking with 5% skim milk for 2 h, the membrane was incubated with a P144 specific monoclonal antibody or a control mouse IgG at a concentration of 1 μg/ml. After washing, the membrane was incubated with HRP conjugated rabbit anti-human IgG antibody (Cat# ab6759, Abcam, UK) or HRP conjugated goat anti-mouse IgG antibody (Cat # 115–035-003, Jackson Immuno Research, USA) diluted 1:5000 in TBST (Tris-buffered saline, pH 8.0, 0.05% Tween 20) containing 5% skim milk. After wash, the bands were developed with an ultra-sensitive ECL substrate (Cat# K-12045-D10, Advansta, USA). The area corresponding to the specific WB bands were excised from the gel stained with Coomassie blue and analyzed using the mass spectrometry.

### Mass spectrometry analysis

The FASP digestion was adapted for the following procedures in Microcon PL-10 filters (Cat#MRCPRT010, Merck, USA). After three-time buffer displacement with 8 M Urea (Cat#U111898, Aladdin, China) and 100 mM Tris-HCl, pH 8.5, proteins were reduced by 10 mM DTT (Cat#646563, Sigma Aldrich, USA) at 37°C for 30 min and followed by alkylation with 30 mM iodoacetamide at 25°C for 45 min in dark. Digestion was carried out with trypsin (enzyme/protein as 1:50) (Cat#T9201, Sigma Aldrich, USA) at 37°C for 12 h after a wash with 20% ACN (Cat#34851, Sigma Aldrich, USA) and three-time buffer displacement with digestion buffer (30 mM Tris-HCl, pH 8.0). After digestion, the solution was filtrated out and the filter was washed twice with 15% ACN, and all filtrates were pooled and vacuum-dried to reach a final concentration to 1 mg/ml. LC-MS analysis was performed using a nanoflow EASYnLC 1200 system (Thermo Fisher Scientific, Odense, Denmark) coupled to an Orbitrap Fusion Lumos mass spectrometer (Thermo Fisher Scientific, Bremen, Germany). A one-column system was adopted for all analyses. Samples were analyzed on a home-made C18 analytical column (75 µm i.d. × 25 cm, ReproSil-Pur 120 C18-AQ, 1.9 µm (Dr. Maisch GmbH, Germany). The mobile phases consisted of Solution A (0.1% formic acid (Cat#695076, Sigma Aldrich, USA) and Solution B (0.1% formic acid in 80% ACN). The derivatized peptides were eluted using the following gradients: 2–5% B in 2 min, 5–35% B in 100 min, 35–44% B in 6 min, 44–100% B in 3 min, 100% B for 10 min, at a flow rate of 200 nl/min. Data-dependent analysis was employed in MS analysis: The time between master scan was 3s, and fragmented in HCD mode, normalized collision energy was 30.

### Construction and preparation of a candidate DNA vaccine encoding SARS-CoV-2 full length S protein

The full-length *s* gene sequence of the reference SARS-CoV-2 Wuhan strain (NCBI accession number: NC_045512.2) was optimized according to the preference of human codon usage and synthesized by GENEWIZ life science company (Suchow, China). The codon optimized spike gene was subcloned into a eukaryotic expression vector (pJW4303, kindly gifted by Dr. Shan Lu’s Laboratory at the University of Massachusetts).^58,59^ And the sequence of inserted gene was verified by Sanger sequencing (Sangon Biotech Co., Ltd., Shanghai, China). The *in vitro* expression of s gene of DNA vaccine was verified by western blot. An EndoFree Plasmid Purification Kit (Cat#12391, Qiagen, Hilden, USA) was used to prepare the recombinant plasmid for mouse vaccination.

### Mouse vaccination

Peripheral blood samples were collected from female adult mice and preexisting S2 binding antibodies were measured using the previously described in-house ELISA method. According to their preexisting S2 binding antibody levels (at 1:100 dilution of serum), the mice were divided into three groups: low (0.015< OD_450nm-630nm_≤0.130, n = 6), moderate (0.130< OD_450nm-630nm_≤0.750, n = 6) and high (OD_450nm-630nm_>0.750, n = 6). All mice were immunized intramuscularly with the candidate S protein DNA vaccine (50 μg/mouse) for three times at an interval of 2 weeks. Three weeks post the third vaccination, the mice were euthanized. Peripheral blood, bronchial lavage and spleen were collected for assays of S protein-specific immune responses.

### Metagenomic and 16s rDNA sequencing of mouse gut microbiota

Metagenomic DNA and 16s rDNA were sequenced by SHANGHAI BIOCHIP CO., LTD. For metagenomic DNA sequencing, bacterial DNA was extracted from fecal samples using a TIANamp Stool DNA Kit (Cat#DP328, TIANGEN, China). Then, a total amount of 1 μg DNA per sample was used as input material for the DNA sample preparations. Metagenomic sequencing libraries were generated using NEBNext® Ultra™ DNA Library Prep Kit for Illumina (Cat#E7103, NEB, USA) following manufacturer’s recommendations. Constructed libraries were analyzed for size distribution by Agilent 2100 Bioanalyzer and quantified using real-time PCR. The clustering of the index-coded samples was performed on a cBot Cluster Generation System. After cluster generation, the library preparations were sequenced on an Illumina Novaseq 6000 platform and paired-end reads were generated.

For 16s rDNA sequencing, fragments of 16s rDNA were amplified with specific barcoded primers 338 F (5’-CCTAYGGGRBGCASCAG-3’) and 806 R (5’-GGACTACNNGGGTATCTAAT-3’). After purification of the PCR product, sequencing libraries were generated using NEBNext® Ultra™ DNA Library Prep Kit for Illumina (Cat#E7654, NEB, USA) following manufacturer’s recommendations. After quality evaluation, the library was sequenced on an Illumina Novaseq 6000 platform and 250bp paired-end reads were generated.

### SARS-CoV-2 pseudo-virus neutralization assay

VSV-backboned SARS-CoV-2 pseudo-viruses were prepared according to a reported method.^60^ The neutralization assay was conducted by following the previously described procedure.^60,61^ Briefly, 100 μl of serially diluted mice sera were added into 96-well cell culture plates. Then, 50 μl of pseudo-viruses with a titer of 13000 TCID_50_/ml were added into each well and the plates were incubated at 37°C for 1 hour. Next, Vero cells were added into each well (2 × 10^4^ cells/well) and the plates were incubated at 37°C in a humidified incubator with 5% CO_2_. 24 hours later, luminescence detection reagent (Bright-Glo™ Luciferase Assay System, Promega, USA) was added to each well following the manufacturer`s instruction. The luminescence was measured using a luminescence microplate reader (GloMax® Navigator Microplate Luminometer, Promega, USA) within 5 minutes. The Reed-Muench method was used to calculate the virus neutralization titer. Antibody neutralization titers were presented as 50% maximal inhibitory concentration (IC_50_).

### Detections of S protein specific cellular immune responses

SARS-CoV-2 S1 and S2 protein-specific IFN-γ releases were measured using the method of enzyme-linked immunosorbent spot (ELISPOT) assays (Cat# 551083, BD Bioscience, USA) according to a previously described procedure.^62^ Briefly, the 96-well ELISPOT plates were coated with purified anti-mouse IFN-γ monoclonal antibody overnight at 4°C. Then, the plates were blocked and 2 × 10^5^ fresh splenocytes were added into each well and incubated with peptide pools of S1 or S2 for 20 hours at 37°C in a humidified incubator with 5% CO_2_. The final concentration for each peptide was 1 μg/ml. After incubation, detecting antibody and Avidin-HRP were added sequentially. Finally, the plates were developed using the BD™ ELISPOT AEC Substrate Set (Cat#551951, BD Bioscience, USA) according to the manufacturer’s manual. Spots representing IFN-γ producing cells were enumerated using an automated ELISPOT plate reader (ChampSpot III Elispot Reader, Saizhi, Beijing, China). At the same time, the supernatants in the wells of ELISPOT plates were also collected for detecting secreted cytokines using a multiplexed cytokine beads array kit (Cat#741054, Biolegend, USA). The concentrations of secreted cytokines were detected using a BD Fortessa flow cytometer (BD Biosciences, USA). Data were analyzed using the LEGENDplex Data Analysis software suit (Biolegend, USA).

### Statistical analysis

All statistical analyses were performed using GraphPad Prism 9 (GraphPad Software, Inc., La Jolla, CA, USA). Normality tests for the data with relatively small sample size were conducted by the method of Shapiro-Wilk test. A method of parametric test was used for the comparisons of normally distributed data and a method of non-parametric test was used for the comparisons of non-normally distributed data. Comparisons between two groups were conducted by the method of *t*-test. Comparisons among three or more group were done using one-way ANOVA. *P* < .05 was considered as statistically significant.

## Supplementary Material

Supplemental Material

## Data Availability

All data needed to evaluate the conclusions in the paper are available in the main text or the supplementary materials. The data of metagenomic analysis of gut microbiota has been deposited to the NCBI Sequence Read Archive (SRA) database with the accession number PRJNA747837. Further information and requests for resources and regents should be contacted with corresponding author Yanmin Wan.
