# Supplementary material for: Preexisting antibodies targeting SARS-CoV-2 S2 cross-react with commensal gut bacteria and impact COVID-19 vaccine induced immunity
Source: Gut Microbes. 2022 Sep 13;14(1):2117503. doi: 10.1080/19490976.2022.2117503 (PMC9481142; doi:10.1080/19490976.2022.2117503)
Supplement: Supplemental Material [file KGMI_A_2117503_SM7005.zip › Supplementary Figures and Figure legends.pdf]

Supplementary figures and figure legends

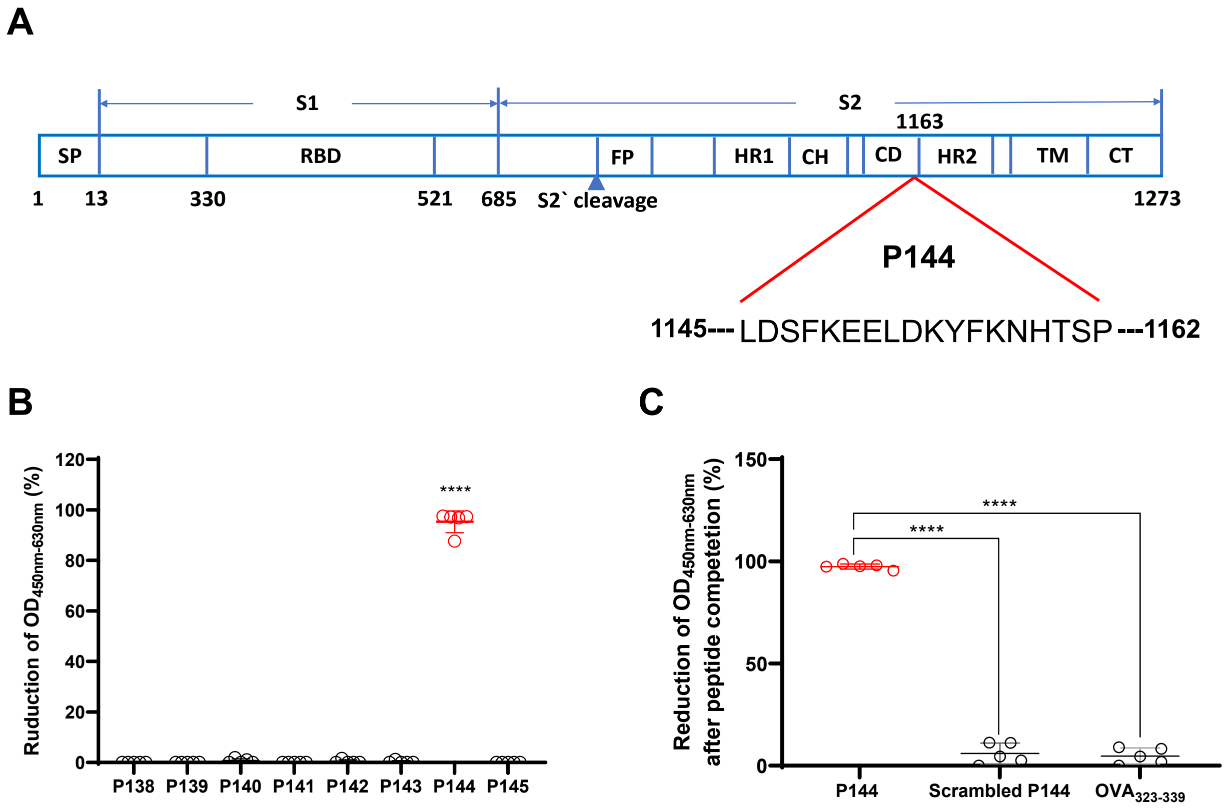

Fig. S1 A dominant linear epitope recognized by the pre-existing antibodies locates on the connector domain of S2. (**A**) Illustration of the location of P144 on the full length of SARS-CoV-2 spike protein. Synthesized peptides (18-mer, overlapped by 11amino acids) spanning the full length of S2 were divided into 9 peptide pools, each contained 8 peptides. To identify the potential antibody binding epitopes, we first performed competitive ELISA assays using the peptide pools as competitors (Data not shown). The pool showing significant inhibition was further delineated by testing the inhibiting efficiencies for each individual peptide. (**B**) A linear antibody epitope was identified via the method of competitive ELISA assay. (**C**) The specificity of P144 mediated inhibition was verified using a scrambled P144 (N`-LKHSKFDLNYKETSDEPF-C`) and a non-relevant peptide (OVA$_{323\text{-}339}$) as controls. The data were shown as mean±SD, n=5. Inhibition efficiencies among groups were compared using the method of One-way ANOVA. ****, $p<0.0001$. SP, signal peptide; RBD, receptor-binding domain; FP, fusion peptide; HR, heptad repeat; CH, central helix; CD, connector domain; TM, transmembrane domain; CT, cytoplasmic tail.

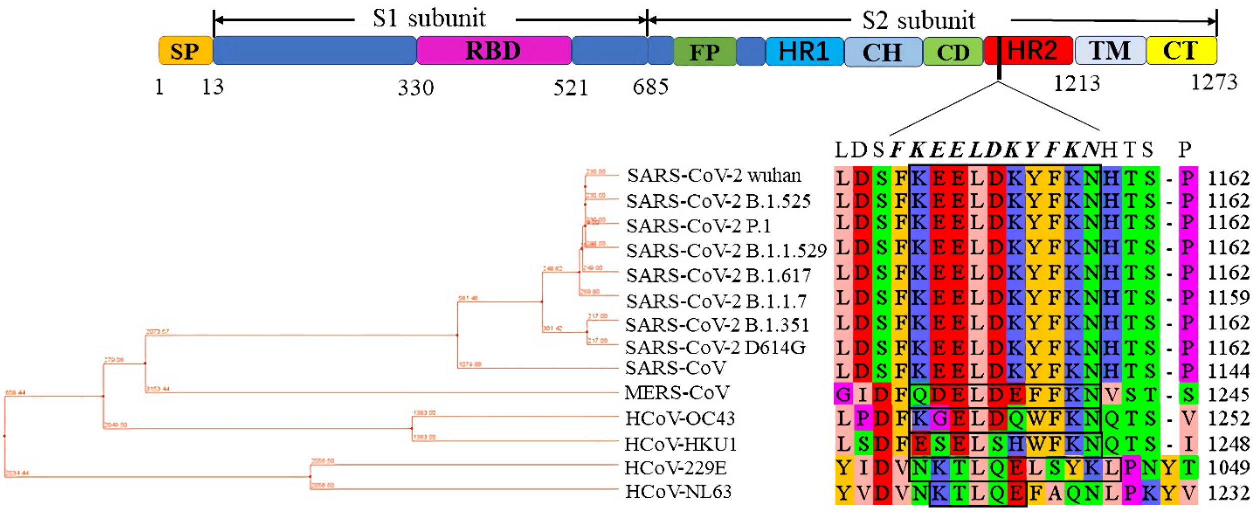

Fig. S2 Phylogenetic analysis of the spike protein sequences of major human coronaviruses. The sequences of SARS-CoV-2 Wuhan (YP_009724390), MERS-CoV (QFQ59587.1), HCoV-OC43 (QDH43719.1), HCoV-NL63 (AKT07952), HCoV-229E (AOG74783.1) and HCoV-HKU1 (YP_173238) were retrieved from NCBI database. The sequences of B.1.1.7, D614G, B.1.351, B.1.525, B.1.617 (Delta), P.1(B.1.1.281) and B.1.1.529 (Omicron) were obtained from Global Initiative on Sharing Avian Influenza Data (GISAID). Boxed fragments represent IEDB predicted linear antibody epitope (http://tools.iedb.org/bcell/).

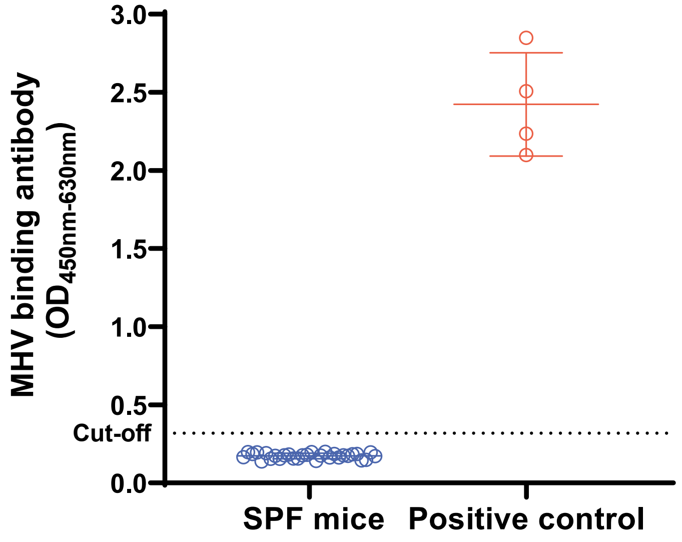

Fig. S3 Detection of MHV binding antibodies in sera of randomly selected mice. Mice from different cages (n=30) were randomly selected for monitoring of MHV infection. The cut-off value was defined as 2-fold of the average OD value of mice in an sterile isolation pack. The positive reference reagent provided in the kit was detected in quadruplicate.

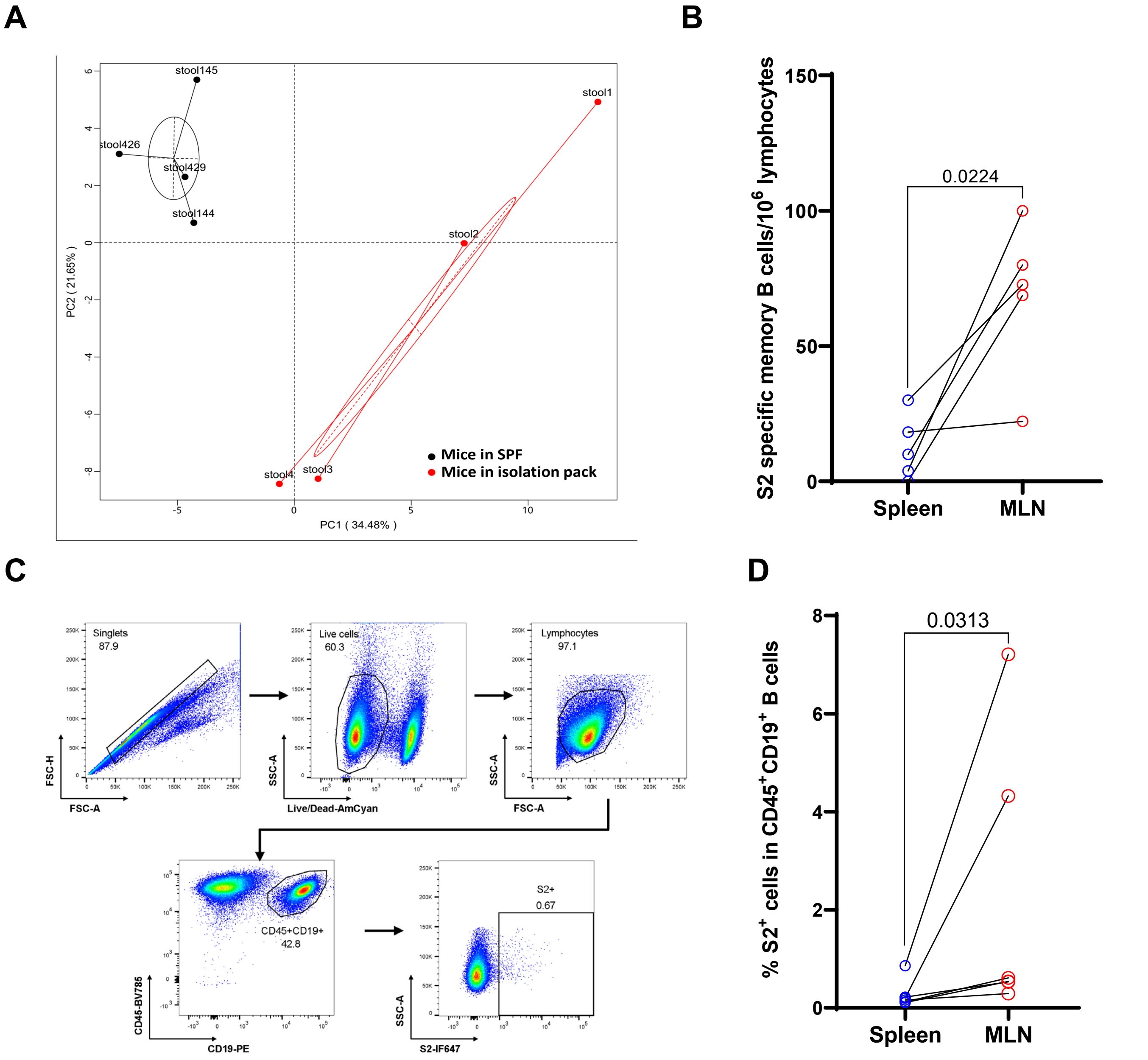

Fig. S4 Evidences implied that pre-existing S2 cross-reactive antibodies were associated with commensal gut bacteria. (**A**) Principle component analysis (PCA) of gut microbial communities between 4 mice in SPF condition and 4 mice in a sterile isolation pack. (**B**) S2 specific memory B cells were measured using the method of memory B cell ELISPOT assay (Cat#3825-2A, Mabtech, Sweden), n=5. Briefly, lymphocytes isolated from mouse mesenteric LN (MLN) or spleen were stimulated with R848 and IL-2. 72 hours later, the cells were washed in DMEM with 10% FBS and then added into a 96-well ELISPOT plate coated with 10μg/ml S2 protein. After being incubated at 37˚C for 20 hours, the plate was washed with PBS, detecting antibody and Streptavidin-Alkaline Phosphatase were added sequentially. Finally, the plate was developed using BCIP/NBT-plus Substrate (Cat#3650-10, Mabtech, Sweden). Spots were enumerated using an automated ELISPOT plate reader. Statistical analysis was performed by the method of paired t-test. (**C**) The gating strategy of the flowcytometry assay. Lymphocytes were isolated from MLNs and spleens of naive C57BL/6J mice with relatively high levels of pre-existing S2 reactive antibodies, n=6. (**D**) The comparison of the frequencies of $S2^{+}CD45^{+}CD19^{+}$ cells between spleens and MLNs. Statistical analysis was performed by the method of paired t-test. Biotin labeled S1 protein was used as a negative control. The experiment was repeated twice.

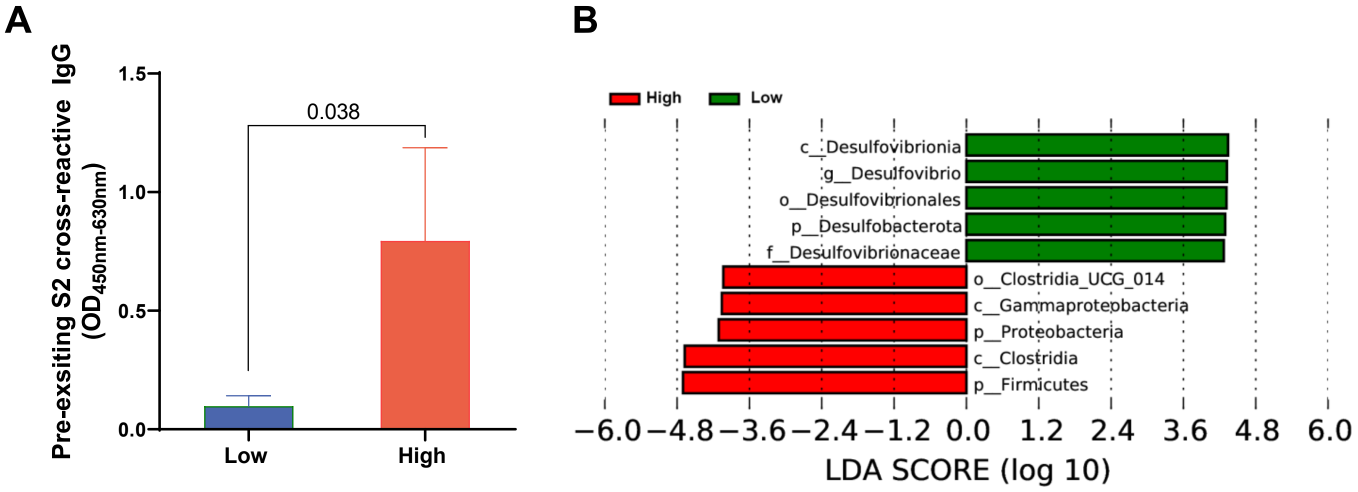

Fig. S5 Comparison of gut microbiota between mice with low or high levels pre-existing S2 reactive antibodies. Peripheral blood and stool samples were collected from 3 mice with high levels of pre-existing S2 reactive antibodies and 3 mice with low levels of pre-existing S2 reactive antibodies. All the mice were housed in cages different from each other. (**A**) The levels of pre-existing S2 reactive antibodies were compared the two groups. (**B**) Commensal gut bacteria compositions were analyzed by 16S rDNA sequencing and compared between the two groups. Bacterial abundances were compared by linear discriminant analysis (LDA) analysis and shown as the histogram of LDA scores. Data in (**A**) were shown as mean±SD, n=3. Statistical analysis for (**A**) was performed by the method of unpaired t-test.

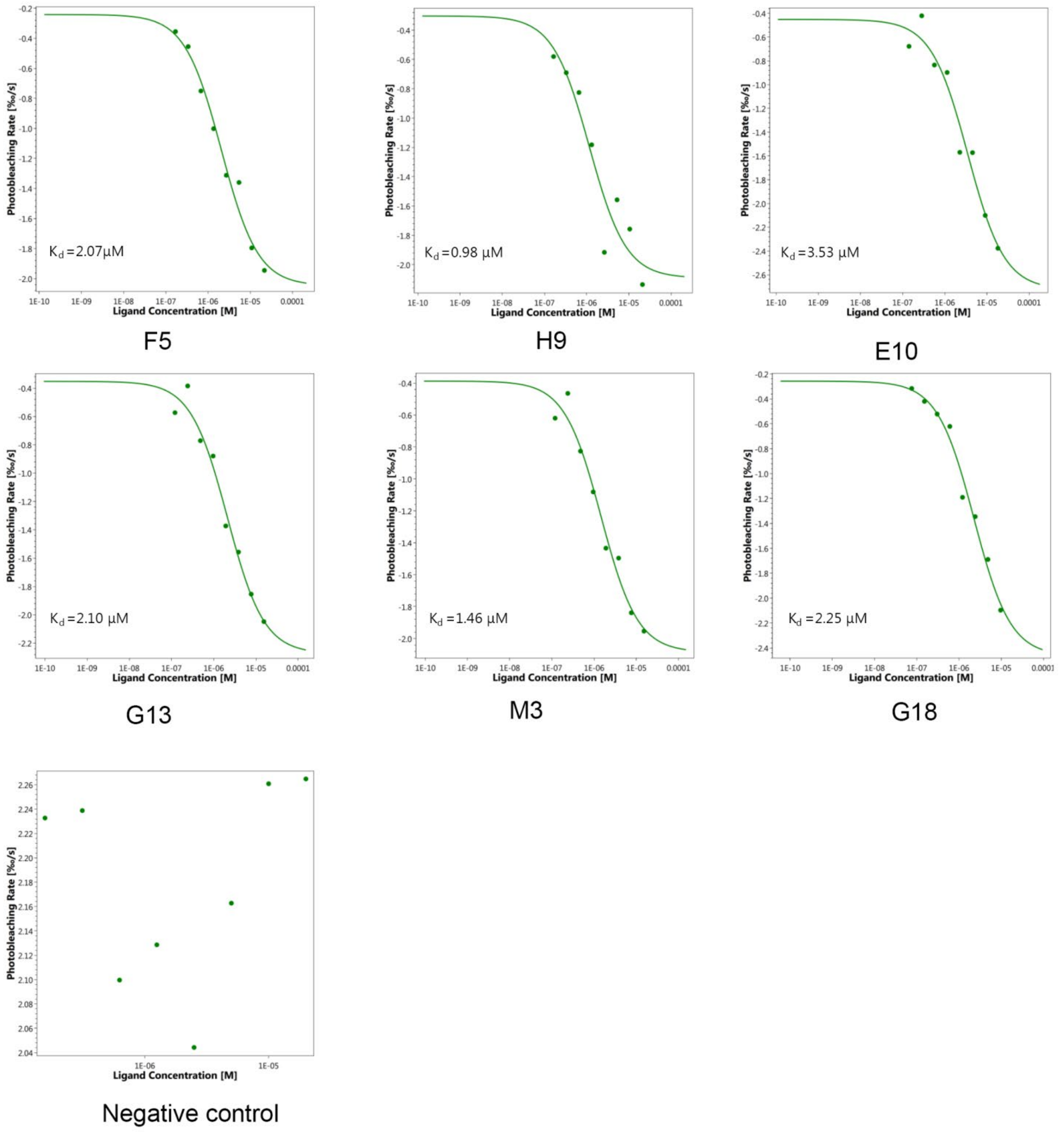

Fig. S6 The binding affinities of the P144 reactive mAbs to S2 protein. The affinities were measured using MicroScale Thermophoresis (Monolith NT.115, Nanotemper, Germany) following the manufacturer`s instruction. Briefly, purified S2 protein (Cat# 40590-V08B，Sino Biological, China) was labeled with the fluorescent dye Red-NHS using Monolith NT protein labeling kit (#MO-L011, Nanotemper, Germany). NHS-Red labeled S2 was incubated with serially diluted mAbs at a final concentration of 5 nM in PBS-T buffer. Five minutes later, the samples were loaded into glass capillaries (Cat#MO-K002, Nanotemper, Germany) and affinity measurements were performed at 37℃. Dose-response curves of 6 mAbs were acquired and the Kd values were calculated by the MO. Affinity software. Uncoupled NHS-Red dye was used as a negative control.

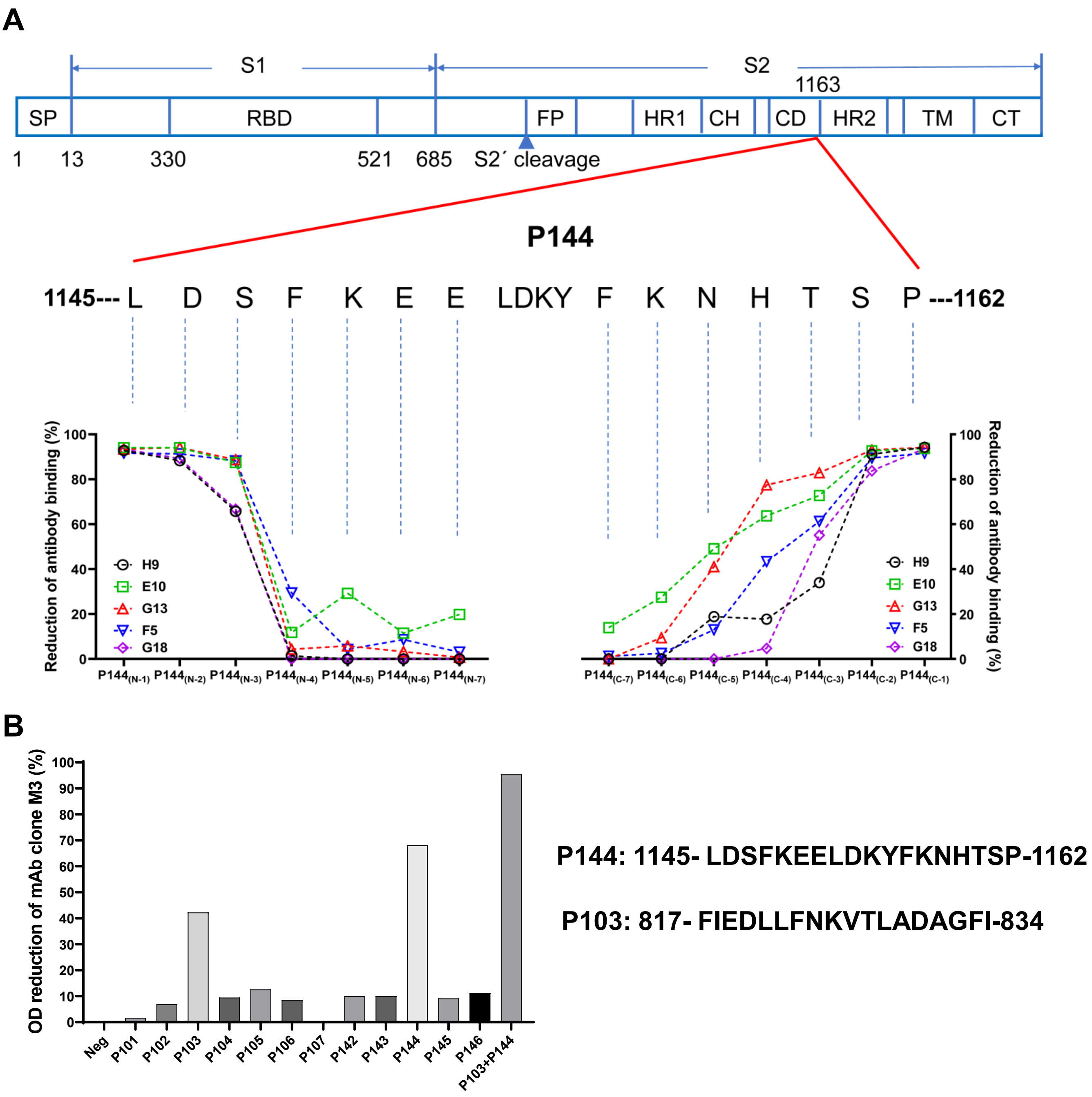

Fig. S7 Characterizations of the minimal epitope recognitions for the 6 monoclonal antibodies isolated from naïve mice. Six P144 specific monoclonal antibodies were isolated from 2 naïve SPF mice using the hybridoma technology. (**A**) The minimum epitope recognitions of clones H9, E10, G13, F5 and G18 were similar detected by a method of competitive ELISA. Purified S2 protein was used as the coating antigen and the truncated peptides derived from P144 were used as the competitors. Reduction of antibody binding was calculated as the percentage of OD reduction upon peptide competition. (**B**) The epitope recognition of clone M3 was analyzed using purified S2 protein as the coating antigen and each listed peptide was used as the competitor. SP, signal peptide; RBD, receptor-binding domain; FP, fusion peptide; HR, heptad repeat; CH, central helix; CD, connector domain; TM, trans-membrane domain; CT, cytoplasmic tail.

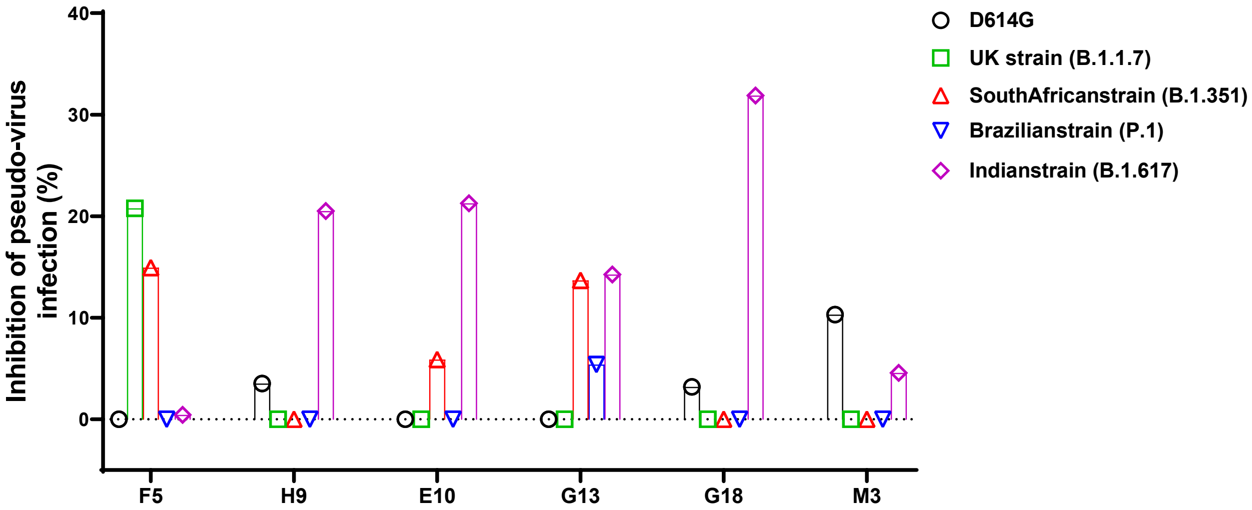

Fig. S8 Neutralizing activities of the purified mAbs as measured using a pseudo-virus neutralization assay. Neutralization activities of 6 isolated mAbs against 5 VSV-backboned SARS-CoV-2 variants (D614G, B.1.617, B.1.1.7, B.1.351 and P.1) pseudo-viruses were examined using a neutralization assay. 50μl of serially diluted monoclonal antibody were added into 96-well cell culture plates. Then, 50μl of pseudo-viruses (650$TCID_{50}$/well) were added into each well. Purified mouse IgG was used as a negative control and serum from an RBD protein vaccinated goat was use as a positive control. Purified mAbs and control mouse IgG were tested at a concentration of 1μg/ml, positive goat serum was diluted at 1:90.

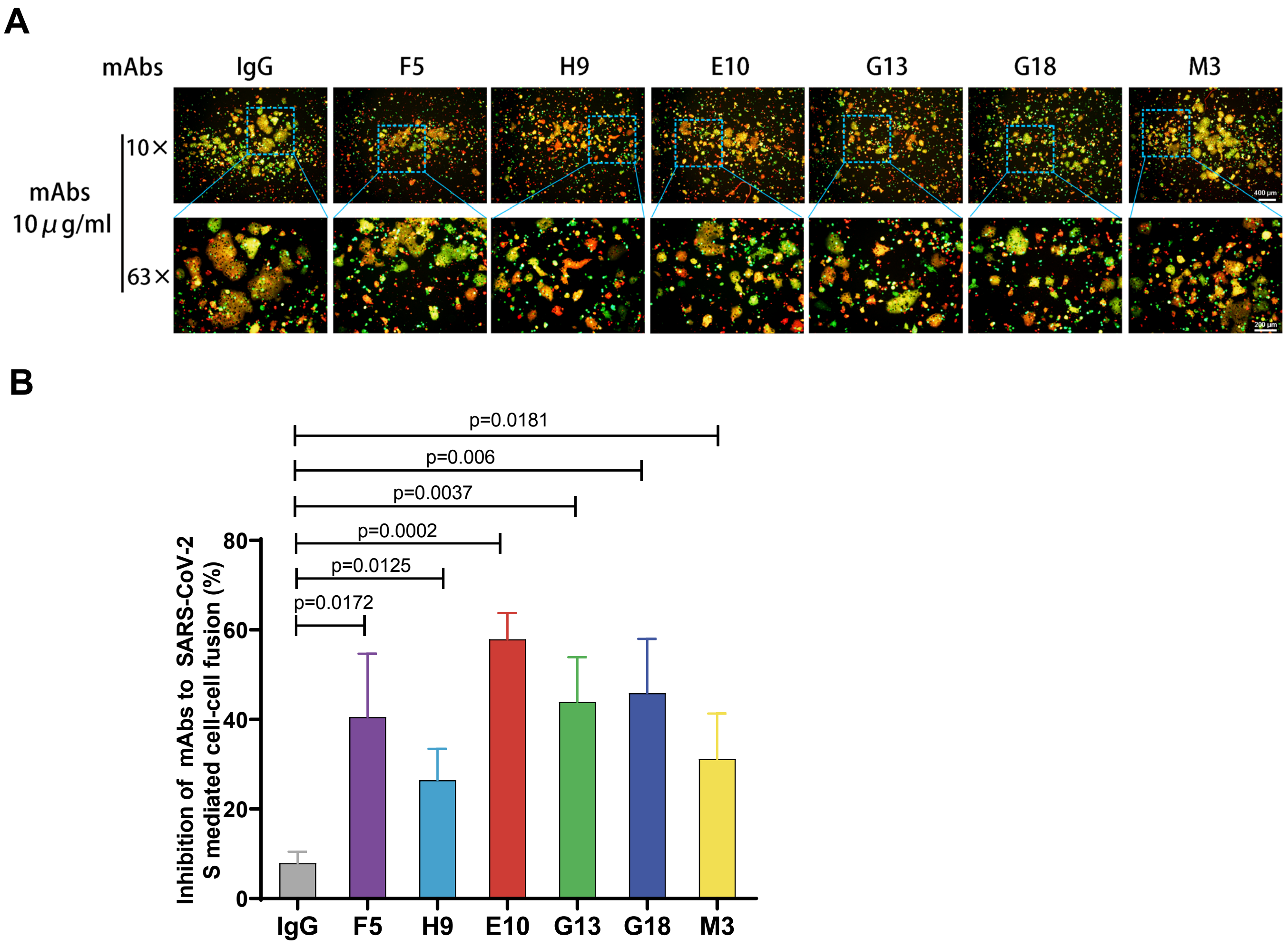

Fig. S9 Inhibition of SARS-CoV-2 spike protein mediated cell fusion by isolated mAbs. HEK293T cells were transiently co-transfected with a plasmid encoding EGFP and a plasmid encoding the full length SARS-CoV-2 spike protein (Wuhan strain). Meanwhile, HEK-293T/hACE2 cells (PackGene Biotech) were transiently transfected with a plasmid encoding mCherry. 48 h later, the cells co-transfected with EGFP and spike plasmids were detached and incubated with an mAb for 1h at 37˚C. Subsequently, these cells were mixed with mCherry transfected 293T/hACE2 cells at a ratio of 1:1 (1×10^5 VS 1×10^5) and plated into a 48-well plate. 4 h later, images of syncytium formation were captured with an Olympus IX73 Inverted LED Fluorescence Microscope. For each test, three fields were randomly selected and imaged. Representative images are shown in (**A**) (the upper panel: 10×; the lower panel: 63×). The yellow cells were counted as fused cells. The percentage of cell-cell fusion was calculated using the following formula: [(number of the yellow cells)/(number of the green cells+yellow cells×100%)]. (**B**) Statistical comparisons between the control IgG and each mAb were done by the method of unpaired t-test.

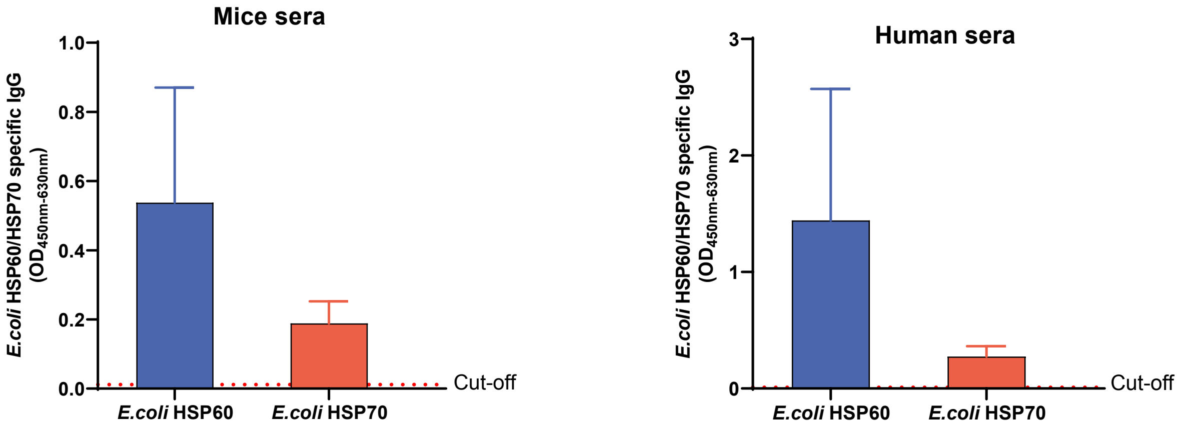

Fig. S10 Detection of HSP60/70 binding antibodies in sera of vaccinated mice and humans. Purified *E.coli* HSP60 and HSP70 proteins were used as the coating antigens. Sera of mice received 3 doses of a DNA vaccine (n=5) and sera of healthy individuals received 2 doses of an inactivated SARS-CoV-2 vaccine (n=6) were diluted at 1:100. Heat inactivated mouse or human sera were used as negative controls. The cut-off values were defined as 2-fold of the average values of negative controls.

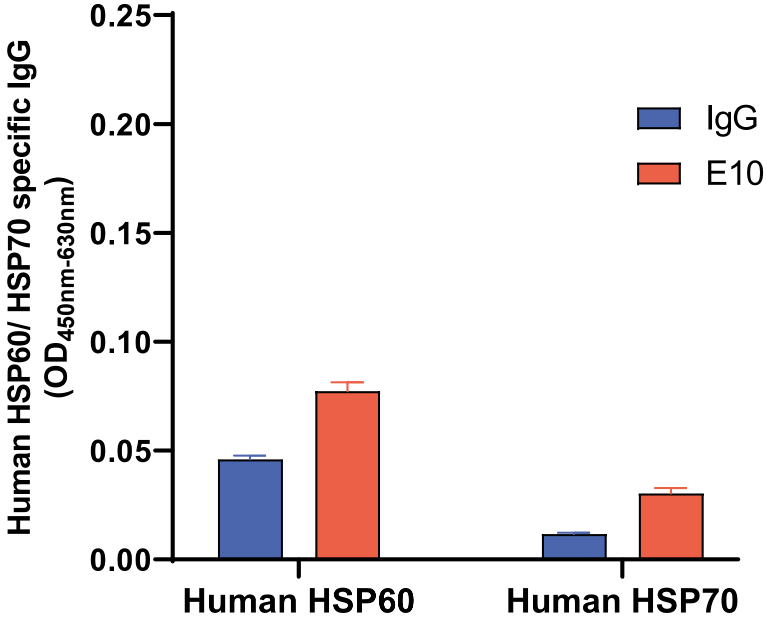

Fig. S11 Recognition of the human HSP60 and HSP70 proteins by a P144 reactive mAb (E10). Purified human HSP60 and HSP70 proteins were used as the coating antigens. E10 or a purified mouse IgG was used as primary antibody at the concentration of 10μg/ml.

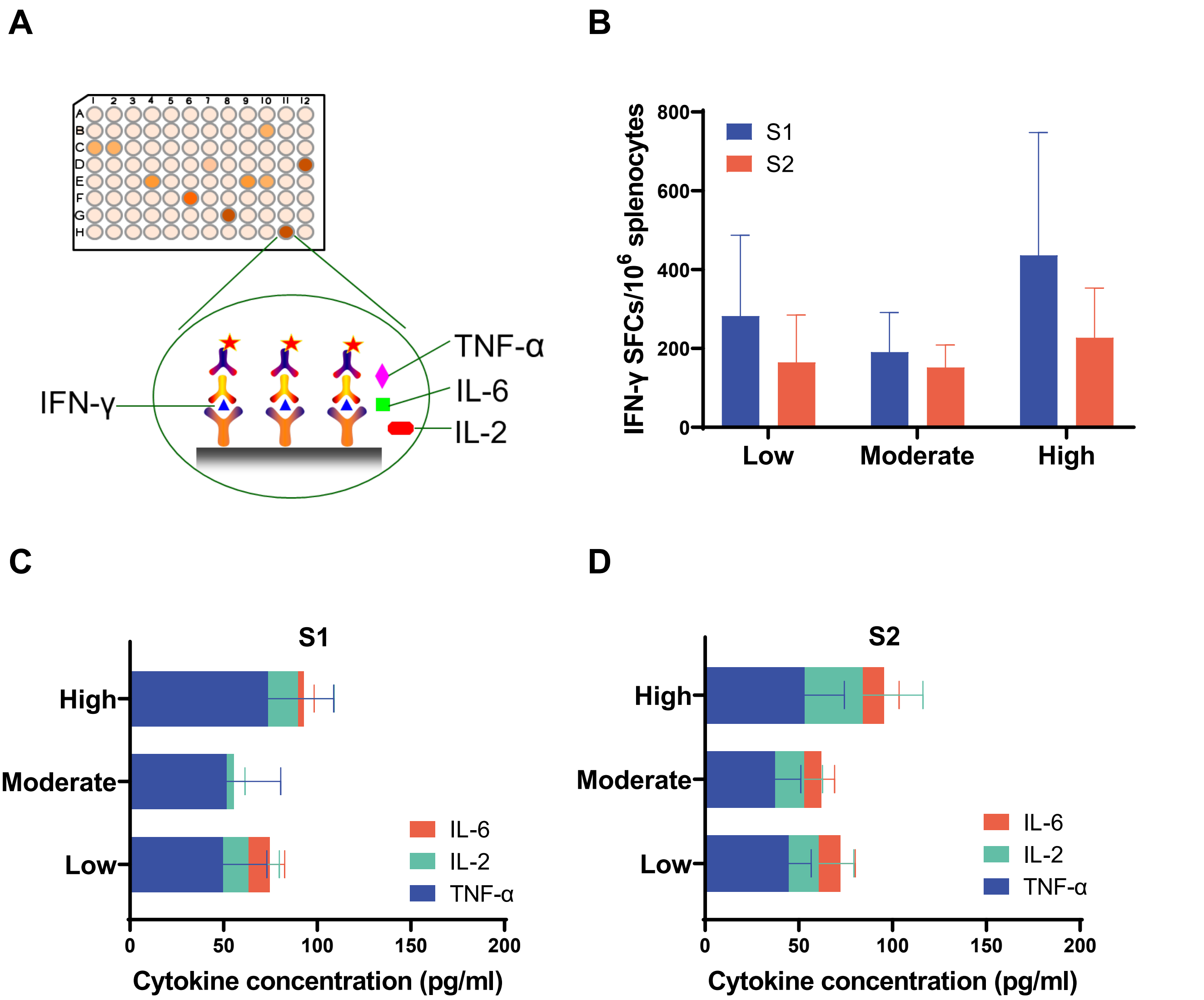

Fig. S12 The impacts of pre-existing antibody on the cellular immune responses after vaccination. (**A**) The diagram of the method used for cellular immune responses evaluation. Antigen specific IFN-γ releases were measured using the method of ELISPOT assay and cytokines in supernatants were detected using the multiplexed cytokine beads assay. (**B**) S1 and S2 specific IFN-γ responses were compared among groups of mice with different levels of pre-existing S2 reactive antibodies. Additionally, S1 (**C**) and S2 (**D**) specific releases of IL-2, IL-6 and TNF-α as measured using the method of multiplex cytokine bead assay were also compared among different groups. Stacked columns represent mean concentrations of TNF-α (Blue), IL-6 (Pink) and IL-2 (Green), respectively. Data were shown as mean± SD, n=6. SFCs, spot forming cells.

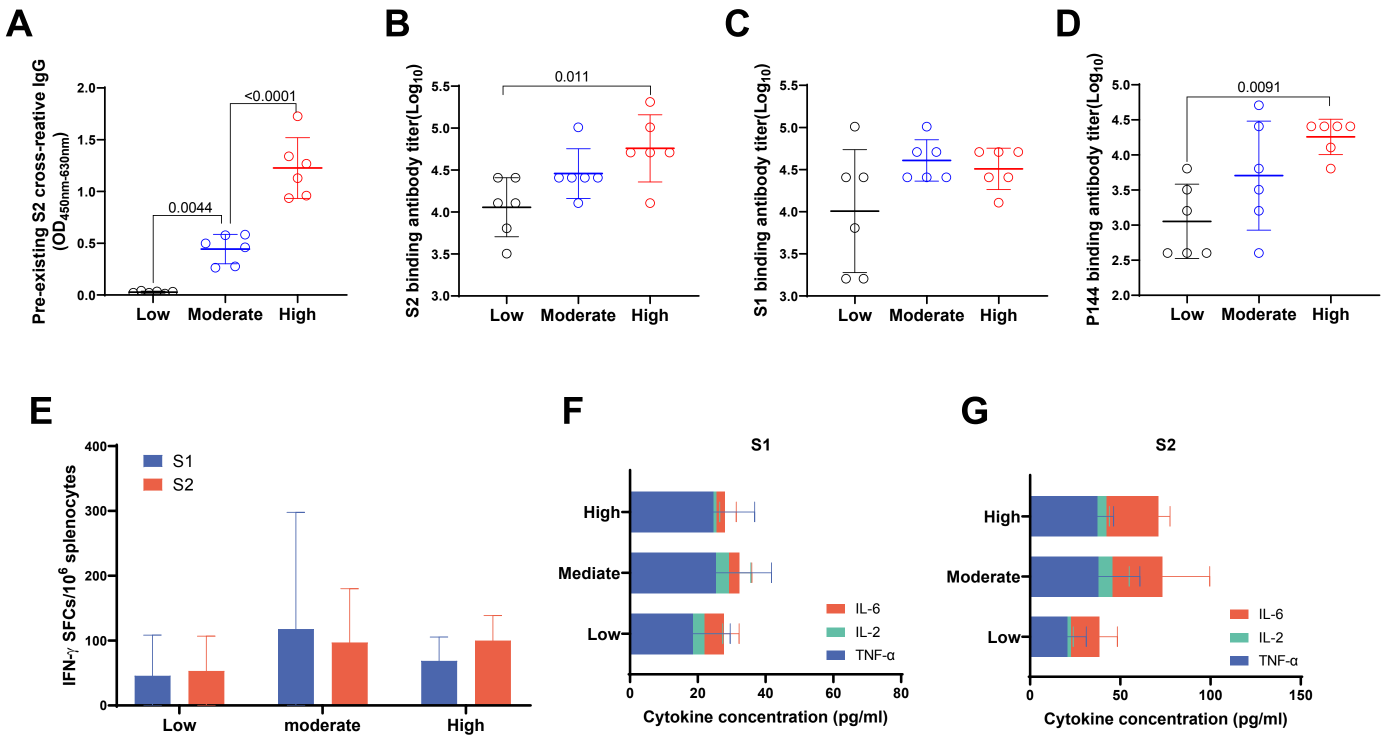

Fig. S13 Impact of pre-existing antibodies on the immune responses elicited by a DNA vaccine encoding SARS-CoV-2 S protein (Validating experiment). 50μg of the DNA vaccine was injected intra muscularly into each mice at week 0, week 2 and week 4, respectively. Two weeks after the final vaccination, the mice was euthanized for the measurements of specific immune responses. (**A**) Peripheral blood was collected before immunization and levels of pre-existing S2 specific IgG were compared among three groups. (**B**) Comparisons of endpoint IgG titers against S2 measured at 2 weeks post the final vaccination. (**C**) Comparisons of endpoint IgG titers against S1 measured at 2 weeks post the last immunization. (**D**) Comparisons of P144 specific IgG titers measured using BSA-P144 conjugate as the coating antigen (**E**) S1 and S2 specific IFN-γ responses were compared among different groups at 2 weeks post the final immunization. S1 (**F**) and S2 (**G**) specific releases of IL-2, IL-6 and TNF-α as measured using the method of multiplex cytokine bead assay were also compared among different groups. Data were shown as mean± SD, n=6. SFCs, spot forming cells. Statistical analyses were performed by the method of one-way ANOVA.
