# Supplementary material for: Preexisting antibodies targeting SARS-CoV-2 S2 cross-react with commensal gut bacteria and impact COVID-19 vaccine induced immunity
Source: Gut Microbes. 2022 Sep 13;14(1):2117503. doi: 10.1080/19490976.2022.2117503 (PMC9481142; doi:10.1080/19490976.2022.2117503)
Supplement: Supplemental Material [file KGMI_A_2117503_SM7005.zip › Supplementary Table 1 (1).docx]

Table S1 V(D)J gene sequencing of P144 reactive mAbs

| **mAb Clone No.** | **Chain type** | **Top V gene match** | **Top D gene match** | **Top J gene match** | **CDR1** | **CDR2** | **CDR3** |
| --- | --- | --- | --- | --- | --- | --- | --- |
| E10 | VH | IGHV5-9*02 | IGHD2-3*01 | IGHJ2*01 | GFAFSSYD | ISSGGSYT | ARQDGYYRYFDY |
|  | VL | IGKV8-30*01 |  | IGKJ1*01 | QSLLYSSNQKNY | WAS | QQYYSYPPT |
| F5 | VH | IGHV2-5*01 | IGHD1-1*01 | IGHJ2*01 | GFSLTSYG | IWRGGST | AKIDGSSNY |
|  | VK | IGKV6-23*01 |  | IGKJ2*01 | QDVGTA | WAS | QQYSSYPT |
| G13 | VH | IGHV2-5*01 | IGHD1-1*01 | IGHJ2*01 | GFSLTSYG | IWRGGST | AKIDGSSNY |
|  | VL | IGLV2*02 |  | IGLJ2*01 | TGAVTTSNY | GTS | ALWYSTHYV |
| G18 | VH | IGHV2-5*01 | IGHD1-1*01 | IGHJ2*01 | GFSLTSYG | IWRGGST | AKIDGSSNY |
|  | VK | IGKV6-23*01 |  | IGKJ2*01 | QDVGTA | WAS | QQYSSYPT |
| H9 | VH | IGHV2-5*01 | IGHD1-1*01 | IGHJ2*01 | GFSLTSYG | IWRGGST | AKIDGSSNY |
|  | VK | IGKV6-23*01 |  | IGKJ2*01 | QDVGTA | WAS | QQYSSYPT |
| M3 | VH | IGHV2-5*01 | IGHD1-1*01 | IGHJ2*01 | GFSLTSYG | IWRGGST | AKIDGSSNY |
|  | VK | IGKV6-23*01 |  | IGKJ2*01 | QDVGTA | WAS | QQYSSYPT |

**Note**: G13,F5 and M3 were isolated from a naïve C57BL/6J mouse; E10,G18 and H9 were isolated from a naïve BALB/c mouse .
