# Supplementary material for: Preexisting antibodies targeting SARS-CoV-2 S2 cross-react with commensal gut bacteria and impact COVID-19 vaccine induced immunity
Source: Gut Microbes. 2022 Sep 13;14(1):2117503. doi: 10.1080/19490976.2022.2117503 (PMC9481142; doi:10.1080/19490976.2022.2117503)
Supplement: Supplemental Material [file KGMI_A_2117503_SM7005.zip › Supplementary Table 2 (1).docx]

Table S2 Potential cross-reactive antigens identified in mouse fecal bacteria

| **NCBI Accession #** | **Protein name** | **Bacterium** | **Score** | **Proteins** | **Unique Peptides** | **Peptides** | **PSMs** | **Area** | **MW [kDa]** |
| --- | --- | --- | --- | --- | --- | --- | --- | --- | --- |
| Q8A470 | DNA-directed RNA polymerase subunit beta' OS | Bacteroides thetaiotaomicron | 168.01 | 550 | 2 | 6 | 6 | 3.536E7 | 158.3 |
| Q5L897 | DNA-directed RNA polymerase subunit beta OS | Bacteroides fragilis | 92.15 | 8 | 2 | 3 | 3 | 8.315E6 | 142.4 |
| Q8A1G1 | TonB-dependent receptor SusC OS | Bacteroides thetaiotaomicron | 145.74 | 1 | 1 | 2 | 3 | 1.385E8 | 111.1 |
| Q46509 | Aldehyde oxidoreductase OS | Desulfovibrio gigas | 75.93 | 1 | 1 | 1 | 1 | 8.506E6 | 97.0 |
| P22983 | Pyruvate, phosphate dikinase OS | Clostridium symbiosum | 109.83 | 7 | 2 | 2 | 2 | 3.721E7 | 96.6 |
| P0A9Q8 | Aldehyde-alcohol dehydrogenase OS | Escherichia coli | 34.29 | 1 | 1 | 1 | 1 | 1.085E7 | 96.1 |
| Q826F6 | Chaperone protein dnaK2 OS | Streptomyces avermitilis | 20.37 | 1 | 1 | 1 | 1 | 1.059E7 | 67.5 |
| Q892R0 | Chaperone protein DnaK OS | Clostridium tetani | 51.52 | 5 | 1 | 1 | 1 | 1.780E7 | 66.4 |
| B1I9W8 | L-fucose isomerase OS | Streptococcus pneumoniae | 57.33 | 12 | 2 | 2 | 2 | 2.792E7 | 65.7 |
| P95334 | Chaperone protein DnaK OS | Myxococcus xanthus | 39.23 | 4 | 1 | 1 | 1 | 1.792E7 | 65.3 |
| Q1MPZ9 | Formate--tetrahydrofolate ligase OS | Lawsonia intracellularis | 62.49 | 1 | 2 | 2 | 2 | 2.068E7 | 64.2 |
| P26929 | Urease subunit alpha OS | Lactobacillus fermentum | 134.06 | 4 | 4 | 4 | 5 | 1.526E7 | 61.8 |
| Q9I165 | Periplasmic trehalase OS | Pseudomonas aeruginosa | 77.05 | 1 | 1 | 1 | 1 | 4.240E8 | 61.1 |
| Q9EZ02 | Pyrophosphate--fructose 6-phosphate 1-phosphotransferase OS | Spirochaeta thermophila | 68.21 | 2 | 1 | 1 | 1 | 1.192E7 | 61.0 |
| P14407 | Fumarate hydratase class I, anaerobic OS | Escherichia coli | 30.79 | 1 | 1 | 1 | 1 | 6.580E6 | 60.1 |
| Q189R2 | Formate--tetrahydrofolate ligase OS | Clostridioides difficile | 123.52 | 22 | 3 | 4 | 4 | 2.621E7 | 59.9 |
| P22252 | Flagellin B OS | Campylobacter jejuni subsp. jejuni serotype O:6 | 75.42 | 1 | 1 | 1 | 1 | 1.478E7 | 59.7 |
| Q1WTW0 | Formate--tetrahydrofolate ligase OS | Lactobacillus salivarius | 83.43 | 23 | 1 | 1 | 1 | 5.229E7 | 59.4 |
| A7HLZ4 | Formate--tetrahydrofolate ligase OS | Fervidobacterium nodosum | 93.49 | 29 | 1 | 2 | 2 | 8.036E6 | 59.2 |
| C4ZBL1 | Phosphoenolpyruvate carboxykinase (ATP) OS | Agathobacter rectalis | 211.34 | 4 | 1 | 4 | 5 | 3.965E7 | 59.0 |
| A6LFQ4 | Phosphoenolpyruvate carboxykinase (ATP) OS | Parabacteroides distasonis | 32.29 | 1 | 1 | 1 | 1 | 8.523E6 | 58.9 |
| A3MZI4 | Formate--tetrahydrofolate ligase OS | Actinobacillus pleuropneumoniae serotype 5b | 78.45 | 78 | 1 | 2 | 2 | 1.320E8 | 58.9 |
| Q2LPJ8 | 60 kDa chaperonin 1 OS | Syntrophus aciditrophicus | 101.22 | 15 | 1 | 2 | 3 | 2.217E7 | 58.6 |
| Q3ALZ3 | 60 kDa chaperonin 1 OS | Synechococcus sp. | 113.34 | 23 | 1 | 2 | 3 | 9.973E7 | 58.5 |
| B8J123 | 60 kDa chaperonin OS | Desulfovibrio desulfuricans | 232.59 | 27 | 2 | 5 | 6 | 9.659E7 | 58.4 |
| Q72AL6 | 60 kDa chaperonin OS | Desulfovibrio vulgaris | 126.02 | 9 | 1 | 3 | 3 | 2.402E7 | 58.4 |
| A0Q2T1 | 60 kDa chaperonin OS | Clostridium novyi | 78.78 | 16 | 1 | 2 | 2 | 2.292E7 | 58.1 |
| B0SCC0 | 60 kDa chaperonin OS | Leptospira biflexa serovar Patoc | 49.58 | 1 | 1 | 1 | 1 | 1.882E8 | 58.1 |
| B2TIX0 | 60 kDa chaperonin OS | Clostridium botulinum | 126.34 | 35 | 1 | 3 | 3 | 4.903E7 | 57.9 |
| A7GZ43 | 60 kDa chaperonin OS | Campylobacter curvus | 105.05 | 41 | 2 | 3 | 3 | 5.327E7 | 57.9 |
| Q67KB8 | 60 kDa chaperonin OS | Symbiobacterium thermophilum | 107.47 | 126 | 1 | 3 | 3 | 9.411E7 | 57.9 |
| B5YDR9 | 60 kDa chaperonin OS | Dictyoglomus thermophilum | 59.61 | 2 | 1 | 1 | 1 | 3.345E7 | 57.9 |
| Q3ADX3 | 60 kDa chaperonin OS | Carboxydothermus hydrogenoformans | 104.07 | 25 | 1 | 2 | 3 | 7.715E7 | 57.6 |
| O50305 | 60 kDa chaperonin OS | Bacillus halodurans | 250.57 | 55 | 3 | 4 | 6 | 3.021E8 | 57.4 |
| P26821 | 60 kDa chaperonin OS | Clostridium perfringens | 131.27 | 36 | 1 | 3 | 3 | 4.609E7 | 57.3 |
| P37282 | 60 kDa chaperonin OS | Lactococcus lactis subsp. lactis | 102.15 | 18 | 1 | 2 | 2 | 4.298E7 | 57.2 |
| C4ZD46 | 60 kDa chaperonin OS | Agathobacter rectalis | 372.10 | 52 | 3 | 5 | 8 | 3.352E8 | 57.1 |
| A6L8C4 | Glucose-6-phosphate isomerase OS | Parabacteroides distasonis | 88.85 | 55 | 1 | 2 | 3 | 5.392E8 | 48.7 |
| P43793 | NADP-specific glutamate dehydrogenase OS | Haemophilus influenzae | 96.32 | 2 | 1 | 2 | 2 | 7.357E7 | 48.6 |
| P00370 | NADP-specific glutamate dehydrogenase OS | Escherichia coli | 161.88 | 1 | 1 | 3 | 4 | 3.280E8 | 48.6 |
| P15111 | NADP-specific glutamate dehydrogenase OS | Salmonella typhimurium | 185.28 | 4 | 1 | 4 | 5 | 1.755E8 | 48.5 |
| P94316 | NAD-specific glutamate dehydrogenase OS | Bacteroides fragilis | 192.93 | 3 | 3 | 5 | 6 | 1.730E8 | 48.4 |
| Q1WSY0 | Enolase OS | Lactobacillus salivarius | 79.89 | 8 | 3 | 3 | 3 | 1.340E7 | 48.0 |
| B7MD95 | Trigger factor OS | Escherichia coli O45:K1 | 78.80 | 10 | 2 | 2 | 2 | 4.344E7 | 47.8 |
| B2GAM0 | Enolase OS | Lactobacillus fermentum | 79.94 | 8 | 2 | 2 | 2 | 1.858E7 | 47.8 |
| Q6MEY2 | Enolase OS | Protochlamydia amoebophila | 112.67 | 1 | 1 | 1 | 1 | 7.493E7 | 47.5 |
| Q0SNH5 | Enolase OS | Borrelia afzelii | 71.86 | 2 | 1 | 1 | 1 | 4.654E7 | 47.4 |
| G3KIM4 | Lactoyl-CoA dehydratase subunit alpha (Fragment) OS | Anaerotignum propionicum | 63.03 | 1 | 1 | 1 | 1 | 1.136E7 | 47.4 |
| Q9I3S1 | Biofilm dispersion protein BdlA OS | Pseudomonas aeruginosa | 59.07 | 22 | 1 | 1 | 1 | 3.282E7 | 46.9 |
| B8J4A8 | Sulfate adenylyltransferase OS | Desulfovibrio desulfuricans | 210.65 | 1 | 5 | 5 | 5 | 2.656E7 | 46.9 |
| A6L3M9 | Enolase OS | Bacteroides vulgatus | 48.62 | 2 | 1 | 2 | 2 | 3.004E7 | 46.7 |
| Q043Z5 | Enolase 1 OS | Lactobacillus gasseri | 289.72 | 4 | 5 | 5 | 5 | 4.189E7 | 46.6 |
| Q1ISS7 | Enolase OS | Koribacter versatilis | 71.57 | 4 | 1 | 1 | 1 | 7.884E6 | 46.5 |
| B8DTI9 | Enolase OS | Bifidobacterium animalis subsp. lactis | 115.71 | 4 | 2 | 2 | 2 | 1.684E7 | 46.4 |
| A7GUR7 | Enolase OS | Bacillus cytotoxicus | 122.68 | 17 | 2 | 2 | 3 | 2.771E7 | 46.4 |
| Q2LR33 | Enolase OS | Syntrophus aciditrophicus | 31.73 | 1 | 1 | 1 | 1 | 4.823E7 | 46.2 |
| Q89Z05 | Enolase OS | Bacteroides thetaiotaomicron | 140.16 | 2 | 2 | 4 | 5 | 3.005E7 | 46.1 |
| B2RLL7 | Enolase OS | Porphyromonas gingivalis | 123.91 | 2 | 2 | 3 | 3 | 2.636E7 | 45.8 |
| Q6MPQ2 | Enolase OS | Bdellovibrio bacteriovorus | 66.50 | 1 | 1 | 1 | 1 | 8.918E6 | 45.7 |
| B0K742 | Serine hydroxymethyltransferase OS | Thermoanaerobacter pseudethanolicus | 85.67 | 92 | 2 | 2 | 2 | 2.140E7 | 45.6 |
| Q5LFT7 | Peptidase T OS | Bacteroides fragilis | 89.56 | 5 | 1 | 1 | 1 | 7.323E7 | 45.5 |
| A9NEA9 | Serine hydroxymethyltransferase OS | Acholeplasma laidlawii | 44.82 | 3 | 1 | 1 | 1 | 5.875E6 | 45.3 |
| Q7MU77 | Phosphoglycerate kinase OS | Porphyromonas gingivalis | 54.70 | 1 | 1 | 1 | 1 | 2.106E7 | 45.0 |
| Q8A753 | Phosphoglycerate kinase OS | Bacteroides thetaiotaomicron | 25.30 | 1 | 1 | 1 | 1 | 1.320E7 | 45.0 |
| A5VHR0 | Phosphopentomutase OS | Lactobacillus reuteri | 159.08 | 1 | 4 | 4 | 4 | 4.364E7 | 44.0 |
| A8EWM4 | Phosphoglycerate kinase OS | Arcobacter butzleri | 45.52 | 1 | 1 | 1 | 1 | 2.577E7 | 43.8 |
| Q042T5 | Elongation factor Tu OS | Lactobacillus gasseri | 400.92 | 36 | 3 | 10 | 13 | 1.184E8 | 43.7 |
| Q74JU6 | Elongation factor Tu OS | Lactobacillus johnsonii | 348.06 | 36 | 2 | 9 | 12 | 1.184E8 | 43.6 |
| Q5L890 | Elongation factor Tu OS | Bacteroides fragilis | 185.88 | 297 | 3 | 5 | 7 | 1.454E8 | 43.6 |
| Q8R603 | Elongation factor Tu OS | Fusobacterium nucleatum subsp. nucleatum | 150.02 | 302 | 1 | 3 | 3 | 6.170E7 | 43.4 |
| B8J1A0 | Elongation factor Tu OS | Desulfovibrio desulfuricans | 139.50 | 64 | 2 | 3 | 3 | 4.287E7 | 43.4 |
| A5VJ92 | Elongation factor Tu OS | Lactobacillus reuteri | 413.79 | 60 | 8 | 11 | 13 | 3.595E8 | 43.4 |

**Note:** *Score:* The Mascot score. Proteins: The total number of proteins contained in the protein group. *Unique* *Peptides:* The total number of peptides unique to the protein group. *Peptides:* The total number of peptides identified from all included searches for the master protein of the protein group. *PSMs:* The total number of peptide-spectrum matches identified from all included searches for the master protein of the protein group. *Area:* The chromatographic peak area was used to characterize the quantitative abundance of protein.*MW(kDa):* The theoretical molecular weight of the protein.
