# Supplementary material for: Preexisting antibodies targeting SARS-CoV-2 S2 cross-react with commensal gut bacteria and impact COVID-19 vaccine induced immunity
Source: Gut Microbes. 2022 Sep 13;14(1):2117503. doi: 10.1080/19490976.2022.2117503 (PMC9481142; doi:10.1080/19490976.2022.2117503)
Supplement: Supplemental Material [file KGMI_A_2117503_SM7005.zip › Supplementary Table 3 (1).docx]

Table S3 Potential cross-reactive antigens identified in human fecal bacteria

| **NCBI Accession #** | **Protein Name** | **Bacterium** | **Score** | **Proteins** | **Unique Peptides** | **Peptides** | **PSMs** | **Area** | **MW [kDa]** |
| --- | --- | --- | --- | --- | --- | --- | --- | --- | --- |
| A6LFK9 | Polyribonucleotide nucleotidyltransferase OS | Parabacteroides distasonis | 62.68 | 3 | 1 | 2 | 2 | 2.332E7 | 82.0 |
| P75764 | Uncharacterized protein YbhJ OS | Escherichia coli | 27.35 | 1 | 1 | 1 | 1 | 3.198E7 | 81.5 |
| Q8A4N6 | Polyribonucleotide nucleotidyltransferase OS | Bacteroides thetaiotaomicron | 226.97 | 23 | 6 | 7 | 7 | 6.947E7 | 78.3 |
| B3DT30 | Elongation factor G OS | Bifidobacterium longum | 236.98 | 64 | 8 | 10 | 11 | 4.407E7 | 78.1 |
| E1WNR6 | Chaperone protein htpG OS | Bacteroides fragilis | 131.15 | 4 | 1 | 2 | 2 | 4.992E7 | 77.9 |
| O31673 | ATP-dependent Clp protease ATP-binding subunit ClpE OS | Bacillus subtilis | 120.19 | 73 | 1 | 3 | 4 | 8.448E7 | 77.9 |
| A7ZSL5 | Elongation factor G OS | Escherichia coli O139:H28 | 133.81 | 92 | 2 | 3 | 4 | 2.888E7 | 77.5 |
| Q5L8A7 | Elongation factor G OS | Bacteroides fragilis | 431.35 | 187 | 3 | 10 | 14 | 1.391E8 | 77.5 |
| A6KYJ7 | Elongation factor G OS | Bacteroides vulgatus | 361.61 | 186 | 2 | 9 | 12 | 1.342E8 | 77.4 |
| A6LFP0 | Methionine--tRNA ligase OS | Parabacteroides distasonis | 65.86 | 8 | 1 | 1 | 1 | 4.772E7 | 77.4 |
| P39396 | Pyruvate/proton symporter BtsT OS | Escherichia coli | 92.20 | 1 | 2 | 2 | 2 | 1.446E7 | 77.3 |
| A9KNK6 | Polyribonucleotide nucleotidyltransferase OS | Lachnoclostridium phytofermentans | 68.87 | 1 | 1 | 1 | 1 | 1.075E8 | 76.9 |
| Q5L6S5 | Elongation factor G OS | Chlamydia abortus | 76.05 | 58 | 1 | 2 | 3 | 1.622E7 | 76.8 |
| Q67JU0 | Elongation factor G OS | Symbiobacterium thermophilum | 147.39 | 86 | 1 | 4 | 5 | 2.631E8 | 76.8 |
| Q5U8S9 | Elongation factor G OS | Staphylococcus intermedius | 98.88 | 65 | 1 | 3 | 4 | 5.177E7 | 76.7 |
| Q8A294 | Putative K(+)-stimulated pyrophosphate-energized sodium pump OS | Bacteroides thetaiotaomicron | 150.65 | 20 | 1 | 3 | 3 | 1.276E8 | 76.5 |
| B9DYA6 | Elongation factor G OS | Clostridium kluyveri | 136.00 | 67 | 1 | 3 | 4 | 8.826E7 | 76.4 |
| Q5WLR5 | Elongation factor G OS | Bacillus clausii | 134.85 | 81 | 1 | 4 | 5 | 1.119E8 | 76.4 |
| Q97I51 | Translation initiation factor IF-2 OS | Clostridium acetobutylicum | 70.53 | 4 | 1 | 1 | 1 | 2.330E8 | 76.3 |
| A0PXU3 | Elongation factor G OS | Clostridium novyi | 114.21 | 67 | 1 | 3 | 4 | 8.905E7 | 76.1 |
| Q18CF4 | Elongation factor G OS | Clostridioides difficile | 174.49 | 59 | 1 | 4 | 5 | 1.288E8 | 75.8 |
| Q8AB53 | Putative glucosamine-6-phosphate deaminase-like protein BT_0258 OS | Bacteroides thetaiotaomicron | 54.10 | 1 | 1 | 1 | 1 | 4.629E6 | 75.2 |
| Q8XJ01 | Penicillin-binding protein 1A OS | Clostridium perfringens | 39.66 | 3 | 1 | 1 | 1 | 1.599E7 | 75.1 |
| A6L7J7 | Threonine--tRNA ligase OS | Bacteroides vulgatus | 91.33 | 9 | 2 | 2 | 2 | 2.440E7 | 74.2 |
| P30539 | 1,4-alpha-glucan branching enzyme GlgB OS | Butyrivibrio fibrisolvens | 30.12 | 1 | 1 | 1 | 1 | 2.287E7 | 73.8 |
| B2TIT5 | Threonine--tRNA ligase OS | Clostridium botulinum | 53.61 | 2 | 1 | 1 | 1 | 1.877E7 | 73.8 |
| P56116 | Chaperone protein HtpG OS | Helicobacter pylori | 38.02 | 12 | 1 | 1 | 1 | 1.269E7 | 71.2 |
| P0A9P7 | ATP-dependent RNA helicase DeaD OS | Escherichia coli O6:H1 | 85.88 | 5 | 3 | 3 | 4 | 1.336E8 | 70.5 |
| P19410 | 3-oxocholoyl-CoA 4-desaturase OS | Clostridium scindens | 63.87 | 1 | 1 | 1 | 1 | 5.844E7 | 70.2 |
| Q8RHJ2 | Putative K(+)-stimulated pyrophosphate-energized sodium pump OS | Fusobacterium nucleatum subsp. nucleatum | 140.41 | 23 | 1 | 3 | 3 | 1.320E8 | 68.9 |
| A5CX56 | Chaperone protein DnaK OS | Vesicomyosocius okutanii subsp. Calyptogena okutanii | 15.38 | 1 | 1 | 1 | 1 | 2.961E7 | 68.7 |
| Q5LG30 | Chaperone protein DnaK OS | Bacteroides fragilis | 418.39 | 33 | 1 | 9 | 11 | 6.399E7 | 68.6 |
| Q89YW6 | Chaperone protein DnaK OS | Bacteroides thetaiotaomicron | 372.24 | 35 | 1 | 7 | 9 | 6.399E7 | 68.3 |
| A6L2X7 | Chaperone protein DnaK OS | Bacteroides vulgatus | 371.71 | 33 | 1 | 8 | 10 | 6.399E7 | 68.3 |
| Q93GF1 | Chaperone protein DnaK OS | Prevotella loescheii | 185.46 | 1 | 4 | 4 | 4 | 3.512E7 | 68.0 |
| A6LGR5 | 4-hydroxy-3-methylbut-2-en-1-yl diphosphate synthase (flavodoxin) OS | Parabacteroides distasonis | 38.34 | 5 | 1 | 1 | 1 | 2.396E7 | 67.9 |
| B8H444 | ATP-dependent zinc metalloprotease FtsH OS | Caulobacter vibrioides | 26.45 | 6 | 1 | 1 | 1 | 1.241E7 | 67.7 |
| A9KIA6 | Aspartate--tRNA(Asp/Asn) ligase OS | Lachnoclostridium phytofermentans | 31.12 | 1 | 1 | 1 | 1 | 8.400E6 | 67.5 |
| P0AG91 | Protein translocase subunit SecD OS | Escherichia coli O157:H7 | 233.70 | 2 | 6 | 6 | 6 | 2.164E7 | 66.6 |
| Q49Y22 | Chaperone protein DnaK OS | Staphylococcus saprophyticus subsp. saprophyticus | 56.64 | 206 | 1 | 2 | 2 | 1.171E7 | 66.5 |
| A6LBU6 | Aspartate--tRNA ligase OS | Parabacteroides distasonis | 88.06 | 49 | 1 | 2 | 2 | 7.901E6 | 66.4 |
| P21332 | Oligo-1,6-glucosidase OS | Bacillus cereus | 53.99 | 2 | 1 | 1 | 1 | 1.391E7 | 66.0 |
| Q8A5W4 | Lysine--tRNA ligase OS | Bacteroides thetaiotaomicron | 154.84 | 2 | 2 | 2 | 2 | 1.304E7 | 65.9 |
| Q8GBW6 | Methylmalonyl-CoA carboxyltransferase 12S subunit OS | Propionibacterium freudenreichii subsp. shermanii | 98.01 | 1 | 1 | 1 | 2 | 1.386E8 | 65.9 |
| Q67S54 | Chaperone protein DnaK OS | Symbiobacterium thermophilum | 96.29 | 225 | 1 | 3 | 3 | 4.528E7 | 65.7 |
| Q9RQ13 | L-fucose isomerase OS | Bacteroides thetaiotaomicron | 181.20 | 7 | 1 | 5 | 7 | 1.038E8 | 65.7 |
| A6L048 | L-fucose isomerase OS | Bacteroides vulgatus | 162.35 | 5 | 2 | 6 | 7 | 3.672E7 | 65.6 |
| Q56403 | V-type ATP synthase alpha chain OS | Thermus thermophilus | 132.50 | 30 | 1 | 1 | 2 | 1.117E7 | 63.6 |
| Q8G7I6 | Glucose-6-phosphate isomerase OS | Bifidobacterium longum | 531.07 | 4 | 14 | 14 | 15 | 3.155E8 | 63.0 |
| Q4JX51 | Glucose-6-phosphate isomerase OS | Corynebacterium jeikeium | 30.85 | 1 | 1 | 1 | 1 | 3.306E7 | 62.1 |
| Q8FZC4 | 2-isopropylmalate synthase OS | Brucella suis biovar 1 | 51.65 | 8 | 1 | 1 | 1 | 1.014E7 | 61.6 |
| A6TGT4 | Glucose-6-phosphate isomerase OS | Klebsiella pneumoniae subsp. pneumoniae | 53.60 | 21 | 1 | 1 | 1 | 3.337E7 | 61.3 |
| P0AG69 | 30S ribosomal protein S1 OS | Escherichia coli O157:H7 | 88.52 | 1 | 2 | 2 | 2 | 8.393E6 | 61.1 |
| Q9EZ02 | Pyrophosphate--fructose 6-phosphate 1-phosphotransferase OS | Spirochaeta thermophila | 110.07 | 2 | 2 | 2 | 2 | 1.245E8 | 61.0 |
| O31716 | Uncharacterized ABC transporter ATP-binding protein YkpA OS | Bacillus subtilis | 48.45 | 1 | 1 | 1 | 1 | 2.259E6 | 61.0 |
| P59173 | Probable 2,3-bisphosphoglycerate-independent phosphoglycerate mutase OS | Leptospira interrogans serogroup Icterohaemorrhagiae serovar Lai | 61.50 | 2 | 1 | 1 | 2 | 6.104E6 | 61.0 |
| P23843 | Periplasmic oligopeptide-binding protein OS | Escherichia coli | 368.94 | 2 | 11 | 11 | 12 | 1.586E8 | 60.9 |
| Q0SQ82 | Formate--tetrahydrofolate ligase OS | Clostridium perfringens | 167.47 | 26 | 1 | 3 | 4 | 6.063E7 | 60.4 |
| P14407 | Fumarate hydratase class I, anaerobic OS | Escherichia coli | 66.46 | 4 | 2 | 2 | 2 | 4.922E7 | 60.1 |
| Q3A9K2 | Formate--tetrahydrofolate ligase OS | Carboxydothermus hydrogenoformans | 110.68 | 55 | 1 | 3 | 3 | 3.356E8 | 60.1 |
| Q251P8 | Formate--tetrahydrofolate ligase 1 OS | Desulfitobacterium hafniense | 72.85 | 55 | 1 | 2 | 2 | 5.742E8 | 60.0 |
| C0QX38 | Formate--tetrahydrofolate ligase OS | Brachyspira hyodysenteriae | 145.04 | 9 | 1 | 2 | 3 | 2.562E7 | 60.0 |
| Q189R2 | Formate--tetrahydrofolate ligase OS | Clostridioides difficile | 157.46 | 1 | 3 | 3 | 3 | 1.347E9 | 59.9 |
| C4ZBG8 | Formate--tetrahydrofolate ligase OS | Agathobacter rectalis | 235.86 | 56 | 4 | 6 | 6 | 3.610E8 | 59.7 |
| A8AQV7 | Phosphoenolpyruvate carboxykinase (ATP) OS | Citrobacter koseri | 104.30 | 44 | 4 | 5 | 5 | 1.300E8 | 59.6 |
| Q24ZZ8 | Formate--tetrahydrofolate ligase 2 OS | Desulfitobacterium hafniense | 79.67 | 55 | 1 | 2 | 2 | 5.193E8 | 59.4 |
| B2RHV8 | Phosphoenolpyruvate carboxykinase (ATP) OS | Porphyromonas gingivalis | 188.39 | 21 | 2 | 5 | 5 | 2.446E8 | 59.4 |
| Q47VD0 | Phosphoenolpyruvate carboxykinase (ATP) OS | Colwellia psychrerythraea | 111.53 | 16 | 1 | 2 | 2 | 1.952E8 | 59.3 |
| A1R7X2 | Arginine--tRNA ligase OS | Paenarthrobacter aurescens | 43.93 | 15 | 1 | 1 | 1 | 2.994E7 | 59.2 |
| Q8A414 | Phosphoenolpyruvate carboxykinase (ATP) OS | Bacteroides thetaiotaomicron | 293.03 | 21 | 3 | 8 | 8 | 3.939E8 | 59.1 |
| C4ZBL1 | Phosphoenolpyruvate carboxykinase (ATP) OS | Agathobacter rectalis | 501.52 | 17 | 5 | 12 | 15 | 4.167E8 | 59.0 |
| Q5L7N5 | Phosphoenolpyruvate carboxykinase (ATP) OS | Bacteroides fragilis | 420.91 | 17 | 1 | 6 | 12 | 2.103E8 | 59.0 |
| A3CL27 | Formate--tetrahydrofolate ligase 1 OS | Streptococcus sanguinis | 145.32 | 20 | 1 | 2 | 3 | 3.296E7 | 59.0 |
| A6LFQ4 | Phosphoenolpyruvate carboxykinase (ATP) OS | Parabacteroides distasonis | 438.19 | 17 | 5 | 11 | 14 | 2.336E8 | 58.9 |
| C4ZAW6 | Dihydroxy-acid dehydratase OS | Agathobacter rectalis | 193.87 | 6 | 5 | 6 | 6 | 2.765E7 | 58.9 |
| B9E299 | Dihydroxy-acid dehydratase OS | Clostridium kluyveri | 85.82 | 2 | 1 | 2 | 3 | 7.244E7 | 58.8 |
| O09460 | Phosphoenolpyruvate carboxykinase (ATP) OS | Anaerobiospirillum succiniciproducens | 214.42 | 21 | 2 | 5 | 5 | 3.702E8 | 58.6 |
| B2TIR2 | Dihydroxy-acid dehydratase OS | Clostridium botulinum | 46.21 | 2 | 1 | 1 | 1 | 3.131E6 | 58.5 |
| B3DTV2 | ATP synthase subunit alpha OS | Bifidobacterium longum | 383.31 | 393 | 9 | 11 | 12 | 5.746E7 | 58.4 |
| B3DRY6 | Bifunctional purine biosynthesis protein PurH OS | Bifidobacterium longum | 204.27 | 2 | 3 | 3 | 3 | 1.243E8 | 58.4 |
| A6LIG0 | 60 kDa chaperonin OS | Parabacteroides distasonis | 325.96 | 2 | 1 | 7 | 10 | 4.117E8 | 58.3 |
| A5N857 | Ribonuclease Y OS | Clostridium kluyveri | 85.68 | 95 | 1 | 3 | 3 | 4.874E7 | 58.3 |
| Q8G3N6 | Inosine-5'-monophosphate dehydrogenase OS | Bifidobacterium longum | 685.57 | 20 | 15 | 16 | 19 | 2.005E8 | 58.2 |
| Q8A6P8 | 60 kDa chaperonin OS | Bacteroides thetaiotaomicron | 591.81 | 1 | 2 | 11 | 15 | 2.794E8 | 58.2 |
| Q5LAF6 | 60 kDa chaperonin OS | Bacteroides fragilis | 690.75 | 1 | 4 | 15 | 20 | 5.301E8 | 58.2 |
| A0Q2T1 | 60 kDa chaperonin OS | Clostridium novyi | 58.42 | 6 | 2 | 2 | 2 | 2.212E8 | 58.1 |
| A6KXA0 | 60 kDa chaperonin OS | Bacteroides vulgatus | 948.24 | 1 | 11 | 22 | 26 | 6.146E8 | 58.1 |

**Note:** *Score:* The Mascot score. Proteins: The total number of proteins contained in the protein group. *Unique* *Peptides:* The total number of peptides unique to the protein group. *Peptides:* The total number of peptides identified from all included searches for the master protein of the protein group. *PSMs:* The total number of peptide-spectrum matches identified from all included searches for the master protein of the protein group. *Area:* The chromatographic peak area was used to characterize the quantitative abundance of protein.*MW(kDa):* The theoretical molecular weight of the protein.
