# Supplementary material for: Preexisting antibodies targeting SARS-CoV-2 S2 cross-react with commensal gut bacteria and impact COVID-19 vaccine induced immunity
Source: Gut Microbes. 2022 Sep 13;14(1):2117503. doi: 10.1080/19490976.2022.2117503 (PMC9481142; doi:10.1080/19490976.2022.2117503)
Supplement: Supplemental Material [file KGMI_A_2117503_SM7005.zip › Supplementary Table 4 (1).docx]

Table S4 Pre and post vaccination antibody responses of 28 healthy vaccinees

| **NO.** | **Post vaccination Neutralizing antibodies (ng/ml)** | **Post vaccination RBD binding antibody titer (Log)** | **Pre-existing S2 binding antibody titer (Log)^♠^** | **Pre-existing BSA-P144 binding antibody titer (Log)^♣^** |
| --- | --- | --- | --- | --- |
| C2 | 0 | 2.431363764 | 2.903089987 | 2.30103 |
| C3 | 0 | 1.954242509 | 2.602059991 | 2 |
| C4 | 0 | 2.431363764 | 3.505149978 | 2.30103 |
| C5 | 0 | 1.954242509 | 2.301029996 | 2 |
| C7 | 0 | 1.954242509 | 2.301029996 | 2 |
| C8 | 7.692307692 | 1.954242509 | 2.602059991 | 2 |
| C9 | 190.8215385 | 2.908485019 | 2.903089987 | 2 |
| C10 | 0 | 1.954242509 | 2.301029996 | 2 |
| C11 | 819.6161538 | 3.385606274 | 3.806179974 | 2.30103 |
| C12 | 25.00923077 | 1.954242509 | 2.602059991 | 2 |
| C14 | 41.14384615 | 1.954242509 | 2.602059991 | 2 |
| C15 | 252.3284615 | 3.385606274 | 2.301029996 | 2 |
| C16 | 819.9984615 | 3.385606274 | 2.602059991 | 2 |
| C17 | 0 | 1.954242509 | 2.602059991 | 2.30103 |
| C18 | 49.34769231 | 1.954242509 | 2.301029996 | 2 |
| C19 | 46.23615385 | 2.431363764 | 2.602059991 | 2 |
| C20 | 82.89076923 | 2.431363764 | 2.301029996 | 2 |
| C23 | 64.88846154 | 2.431363764 | 3.505149978 | 2 |
| C24 | 96.24692308 | 2.908485019 | 3.505149978 | 2.60206 |
| C25 | 76.72846154 | 2.908485019 | 2.602059991 | 2.30103 |
| C26 | 74.09615385 | 2.908485019 | 2.903089987 | 2.30103 |
| C27 | 43.59692308 | 2.431363764 | 2.602059991 | 2.30103 |
| C28 | 0 | 1.954242509 | 2.301029996 | 2 |
| C30 | 305.9192308 | 3.385606274 | 2.903089987 | 2.60206 |
| C31 | 125.2430769 | 2.908485019 | 2.602059991 | 2.60206 |
| C32 | 30.08615385 | 2.908485019 | 3.505149978 | 2.60206 |
| C35 | 173.7146154 | 2.908485019 | 2.301029996 | 2 |
| C36 | 318.0553846 | 3.862727528 | 2.903089987 | 2.30103 |

Note: Heat denatured human sera were used as negative control. “♠”, Pre-existing S2 binding antibody titers positively correlated with the post vaccination RBD binding antibody titers (r=0.468, p=0.012). “♣”, Pre-existing BSA-P144 binding antibody titers positively correlated with the post vaccination RBD binding antibody titers (r=0.497, p=0.007). Statistical analyses were performed using the method of Spearman’s correlation**.**
