# Supplementary material for: Preexisting antibodies targeting SARS-CoV-2 S2 cross-react with commensal gut bacteria and impact COVID-19 vaccine induced immunity
Source: Gut Microbes. 2022 Sep 13;14(1):2117503. doi: 10.1080/19490976.2022.2117503 (PMC9481142; doi:10.1080/19490976.2022.2117503)
Supplement: Supplemental Material [file KGMI_A_2117503_SM7005.zip › Supplementary Table 5 (1).docx]

Table S5 Demographics of SARS-CoV-2 unexposed healthy individuals

| [**Sample**](http://www.baidu.com/link?url=Gz66mtDL2pa--Y_viXpFAuvjTv2bbHaxvc9s64YsUHgi8Nact-KPpXxA3TmaTSB-xxQXdef7cZ94ayihzQeMKw90msTb1nQv7vW9tsm0RUK) **Collection Time** | **2016** | **2020** |
| --- | --- | --- |
| Number of individuals | 78 | 95 |
| Gender (males, females) | 17, 61 | 78, 17 |
| Age, years (mean ± SD) | 35.88 ± 8.40 | 30.40 ± 7.55 |
